# Genetic analysis and natural history of Parkinson’s disease due to the *LRRK2* G2019S variant

**DOI:** 10.1093/brain/awae073

**Published:** 2024-05-28

**Authors:** Matthew J Kmiecik, Steven Micheletti, Daniella Coker, Karl Heilbron, Jingchunzi Shi, Keaton Stagaman, Teresa Filshtein Sonmez, Pierre Fontanillas, Suyash Shringarpure, Madeleine Wetzel, Helen M Rowbotham, Paul Cannon, Janie F Shelton, David A Hinds, Joyce Y Tung, 23andMe Research Team, Adam Auton, Elizabeth Babalola, Robert K Bell, Jessica Bielenberg, Johnathan Bowes, Katarzyna Bryc, Ninad S Chaudhary, Sayantan Das, Emily DelloRusso, Sarah L Elson, Nicholas Eriksson, Will Freyman, Julie M Granka, Alejandro Hernandez, Barry Hicks, Ethan M Jewett, Yunxuan Jiang, Katelyn Kukar, Alan Kwong, Keng-Han Lin, Bianca A Llamas, Maya Lowe, Matthew H McIntyre, Meghan E Moreno, Priyanka Nandakumar, Dominique T Nguyen, Jared O'Connell, Aaron A Petrakovitz, G David Poznik, Alexandra Reynoso, Morgan Schumacher, Leah Selcer, Anjali J Shastri, Qiaojuan Jane Su, Susana A Tat, Vinh Tran, Xin Wang, Wei Wang, Catherine H Weldon, Peter Wilton, Corinna D Wong, Michael V Holmes, Stella Aslibekyan, Lucy Norcliffe-Kaufmann

**Affiliations:** 23andMe, Inc., Research, Sunnyvale, CA 94086, USA; 23andMe, Inc., Research, Sunnyvale, CA 94086, USA; 23andMe, Inc., Research, Sunnyvale, CA 94086, USA; 23andMe, Inc., Research, Sunnyvale, CA 94086, USA; 23andMe, Inc., Research, Sunnyvale, CA 94086, USA; 23andMe, Inc., Research, Sunnyvale, CA 94086, USA; 23andMe, Inc., Research, Sunnyvale, CA 94086, USA; 23andMe, Inc., Research, Sunnyvale, CA 94086, USA; 23andMe, Inc., Research, Sunnyvale, CA 94086, USA; 23andMe, Inc., Research, Sunnyvale, CA 94086, USA; 23andMe, Inc., Research, Sunnyvale, CA 94086, USA; 23andMe, Inc., Research, Sunnyvale, CA 94086, USA; 23andMe, Inc., Research, Sunnyvale, CA 94086, USA; 23andMe, Inc., Research, Sunnyvale, CA 94086, USA; 23andMe, Inc., Research, Sunnyvale, CA 94086, USA; 23andMe, Inc., Research, Sunnyvale, CA 94086, USA; 23andMe, Inc., Research, Sunnyvale, CA 94086, USA; 23andMe, Inc., Research, Sunnyvale, CA 94086, USA

**Keywords:** Parkinson’s disease, LRRK2 G2019S, polygenic risk score, natural history, movement disorders, REM sleep behaviour disorder

## Abstract

The *LRRK2 G2019S* variant is the most common cause of monogenic Parkinson’s disease (PD); however, questions remain regarding the penetrance, clinical phenotype and natural history of carriers. We performed a 3.5-year prospective longitudinal online study in a large number of 1286 genotyped *LRRK2 G2019S* carriers and 109 154 controls, with and without PD, recruited from the 23andMe Research Cohort.

We collected self-reported motor and non-motor symptoms every 6 months, as well as demographics, family histories and environmental risk factors. Incident cases of PD (phenoconverters) were identified at follow-up. We determined lifetime risk of PD using accelerated failure time modelling and explored the impact of polygenic risk on penetrance. We also computed the genetic ancestry of all *LRRK2 G2019S* carriers in the 23andMe database and identified regions of the world where carrier frequencies are highest.

We observed that despite a 1 year longer disease duration (*P* = 0.016), *LRRK2 G2019S* carriers with PD had similar burden of motor symptoms, yet significantly fewer non-motor symptoms including cognitive difficulties, REM sleep behaviour disorder (RBD) and hyposmia (all *P-*values ≤ 0.0002). The cumulative incidence of PD in *G2019S* carriers by age 80 was 49%. *G2019S* carriers had a 10-fold risk of developing PD versus non-carriers. This rose to a 27-fold risk in *G2019S* carriers with a PD polygenic risk score in the top 25% versus non-carriers in the bottom 25%. In addition to identifying ancient founding events in people of North African and Ashkenazi descent, our genetic ancestry analyses infer that the *G2019S* variant was later introduced to Spanish colonial territories in the Americas. Our results suggest *LRRK2 G2019S* PD appears to be a slowly progressive predominantly motor subtype of PD with a lower prevalence of hyposmia, RBD and cognitive impairment. This suggests that the current prodromal criteria, which are based on idiopathic PD, may lack sensitivity to detect the early phases of *LRRK2* PD in *G2019S* carriers.

We show that polygenic burden may contribute to the development of PD in the *LRRK2 G2019S* carrier population. Collectively, the results should help support screening programmes and candidate enrichment strategies for upcoming trials of *LRRK2* inhibitors in early-stage disease.

## Teaser

The *LRRK2 G2019S* variant is the most common cause of monogenic Parkinson’s disease. In a prospective longitudinal study, Kmiecik *et al.* find that genotyped *LRRK2 G2019S* carriers report a similar burden of motor symptoms to non-carriers with Parkinson’s disease, but fewer non-motor symptoms including cognitive difficulties and hyposmia.

## Introduction

Gain-of-function variants in the leucine-rich repeat kinase 2 gene (*LRRK2*, *PARK8*) are associated with monogenic Parkinson’s disease (PD). The *LRRK2 G2019S* founder mutation is the most common cause of monogenic PD, with a prevalence estimate in the USA of up to 2.1%,^1^ an autosomal dominant pattern of inheritance and high but incomplete penetrance.^2^ The variant is most prevalent in Ashkenazi Jews and North African Berbers, where it is assumed that multiple founding events contributed to the high carrier rate in the two ancestral populations.^3,4^ The LRRK2 cytoplasmic protein has a large and complex structure that functions as both a kinase and GTPase.^5^ The *G2019S* missense variant, although located within the kinase domain,^2,6^ is thought to influence both enzymatic activities.^7^ Increased LRRK2 kinase activity has also been observed in post-mortem brain tissue in idiopathic PD,^8^ leading to the hypothesis that LRRK2 inhibition might present a promising therapeutic strategy for PD in general.

The natural history of PD in *LRRK2* carriers is not well understood due to small sample sizes and the lack of compatible prospective datasets. The lifetime prevalence of PD in *LRRK2 G2019S* carriers is hotly debated, with penetrance estimates ranging from 15% to 80%.^1,9^ Precise phenoconversion rates have not been established.^10^ Validating sensitive and specific prodromal criteria to detect the early phases of *LRRK2* PD, as the symptoms of neurodegeneration are emerging, will be important to design enriched neuroprotective trials in at-risk carriers.^11,12^ As it is not known which combination of symptoms are the first to emerge, it is unclear if the prodromal criteria derived from idiopathic PD apply to patients with *LRRK2* PD,^13^ as these heavily weight both REM sleep behaviour disorder (RBD) and hyposmia as high risk markers.^14,15^ Yet, small cohort studies in *LRRK2 G2019S* carriers with PD suggest that RBD is less prevalent^16^ and reports of anosmia are mixed, with some *LRRK2* PD patients having preserved olfaction.^13,17^

In addition, the clinical course of *LRRK2* PD may progress differently. Observations suggest that *LRRK2* PD is a slowly progressive predominantly motor subtype of PD,^13,18^ with low rates of dementia^19^ and better survival.^20^ While patients with idiopathic PD that develop dementia have the hallmark misfolded α-synuclein-containing intraneuronal Lewy body inclusions throughout the limbic system and neocortex at autopsy,^21^ at least one-third of *LRRK2 G2019S* carriers with PD do not exhibit this classic Lewy body pathology anywhere in the brain, including in the substantia nigra.^19^ More recent studies show that unlike cases of idiopathic PD, *LRRK2* carriers are more likely to have a negative CSF α-synuclein seeding amplification assay test, in which the α-synuclein from *LRRK2* patients does not misfold and promote fibrillization.^12^ Taken together, these studies indicate a difference in the core pathophysiology of *LRRK2* PD, where at least in a subset of patients, their neuronal injury appears to be independent of α-synuclein.

The 23andMe, Inc. research participant database is the largest pre-existing genetic cohort developed via direct-to-consumer genetic testing that contains data on human disease, and is uniquely poised to understand *LRRK2* PD. In 2018, we began an initiative to enrol genotyped *LRRK2 G2019S* carriers into a prospective online natural history study. We compared phenotypic and genotypic differences between carriers and non-carriers with and without PD. We used genetic ancestry to map where the founder mutation arose and understand the migratory patterns. We prospectively identified incident cases and used survival analyses to estimate cumulative incidence of PD among carriers and non-carriers. In addition, we investigated the impact of polygenic risk scores (PRS) on susceptibility to PD in the context of *LRRK2 G2019S*. Currently, these results provide the largest prospective natural history study of *LRRK2 G2019S* PD that spans geographical ancestry, phenotypic and genotypic features.

## Materials and methods

### Participants

23andMe’s Parkinson’s Impact Project (PIP) was launched in 2018 with the goal of conducting an online survey-based longitudinal prospective study of *LRRK2 G2019S* carriers. Recruitment emails explaining the purpose of the study were sent to all eligible *LRRK2 G2019S* carriers from the 23andMe research cohort. All study research participants were >18 years old, US residents, and provided informed consent to volunteer to participate (protocol approval: AAHRPP-accredited Salus IRB). Specific eligibility criteria for the *LRRK2 G2019S* participants included: (i) confirmation of at least one *LRRK2 G2019S* allele; (ii) being aware of their carrier status, i.e. having opted into receiving and opening their 23andMe FDA-cleared *LRRK2 G2019S* carrier status report that included a referral for genetic counselling; and (iii) permission to be recontacted about research opportunities. Recruitment into the cohort continued on a rolling basis. Eligible non-carriers were randomly selected from the 23andMe research cohort. Participants included in the analysis were enrolled between November 2018 and 4 October 2022.

### Study design

Surveys were administered through the 23andMe website and/or mobile application. The baseline survey surfaced to carriers and non-carriers captured: (i) demographics (age, sex, education); (ii) PD diagnosis and family history; (iii) motor symptoms; (iv) mood/depression; (v) cognition/memory; (vi) sleep; (vi) hyposmia; (vii) autonomic symptoms; and (viii) lifestyle/environmental exposures. To capture symptom progression, *LRRK2 G2019S* carriers were recontacted every 6 months for 3.5 years (i.e. seven follow-up surveys, see Supplementary Table 1). Non-carriers were recontacted every 12 months to complete a health update survey and report any new PD diagnosis. Phenoconverters (i.e. incident cases) were defined as carrier and non-carrier participants that reported ‘no’ to a PD diagnosis at baseline (study entry), but subsequently reported ‘yes’ to a new PD diagnosis at any subsequent encounter (>7 days after baseline completion).

### Measures

#### Genetic data

DNA extraction and genotyping were performed on saliva samples by Clinical Laboratory Improvement Amendments-certified and College of American Pathologists-accredited clinical laboratories of Laboratory Corporation of America. Samples were genotyped on one of five genotyping platforms (Supplementary material). The accuracy of genotyping the *LRRK2 G2019S* and *GBA N370S* variants was 99% (97%–100%) with >99% reproducibility and repeatability.^22^*LRRK2 G2019S* carrier status was established by the presence of the genotyped *G2019S* variant.

#### Motor and cognitive symptoms

Motor deficits were assessed by asking participants to self-report having tremor, imbalance/falls and gait changes, including: bradykinesia (walking more slowly), shuffling (taking smaller steps), festination (steps becoming faster and faster when walking), freezing of gait (feet getting stuck/glued to the floor), dragging the feet, and reduced arm swing (swing the arms less). Additional self-reported motor symptoms included stooped posture, hypophonia (softer/quieter speech) and deterioration in handwriting. Cognition and executive functioning were assessed with self-reported difficulties with attention/concentrating, distractibility, multitasking, completing tasks, or understanding over the last 12 months. Diagnosis of mild cognitive impairment (MCI) was collected. Memory was assessed by self-reported worsening of forgetfulness, word recall, remembering the date or misplacing things over the last 12 months. Depression was assessed using a modified version of the Patient Health Questionnaire-9 (PHQ-9).^23^

#### Prodromal markers

We followed the Movement Disorders Society Research Criteria for signs and symptoms of prodromal PD, using proxy answers when needed.^15^ The presence of dream re-enactment behaviour was assessed by asking participants whether they reported acting out dreams or had previously been diagnosed with RBD. Olfaction was assessed by asking participants to rate their current ability to smell, with those that reported ‘very poor’ or ‘poor’ smell being classified as having possible hyposmia. Autonomic symptoms were assessed over the last 3 months and included orthostatic hypotension (light headedness), constipation (<3 bowel movements/week), urinary dysfunction (urgency or frequency), and erectile dysfunction. Daytime somnolence was derived from the PHQ-9 in participants that reported more than half of the time trouble falling/staying asleep, sleeping too much, feeling tired or having little energy.

#### Risk markers

Six questions were administered to assess lifetime environmental exposures to pesticides/solvents/heavy metals in the workplace/home and employment requiring the use of a respirator, mask or gloves. Occupational toxin exposure was classified as responding ‘yes’ to regular exposure to pesticides, including herbicides, fungicides, insecticides, rodenticides or fumigants at work. Occupational PPE (personal protective equipment) use was classified as responding ‘yes’ to ever having regularly used a respirator, mask or gloves at the workplace. Participants that did not report weekly consumption of coffee were classified as low-caffeine intake. Following the established standardized definition,^24^ non-smoking status was determined as smoking <100 cigarettes in a lifetime. Physical inactivity was determined as participants that reported they did not participate weekly in any form of physical activity. Family history of PD was defined as having a first-degree relative with PD. Lifetime history of head injury with loss of consciousness due to sports, accidents or violence was also captured.

### Data analysis

#### Baseline comparisons

**Figure 1:**
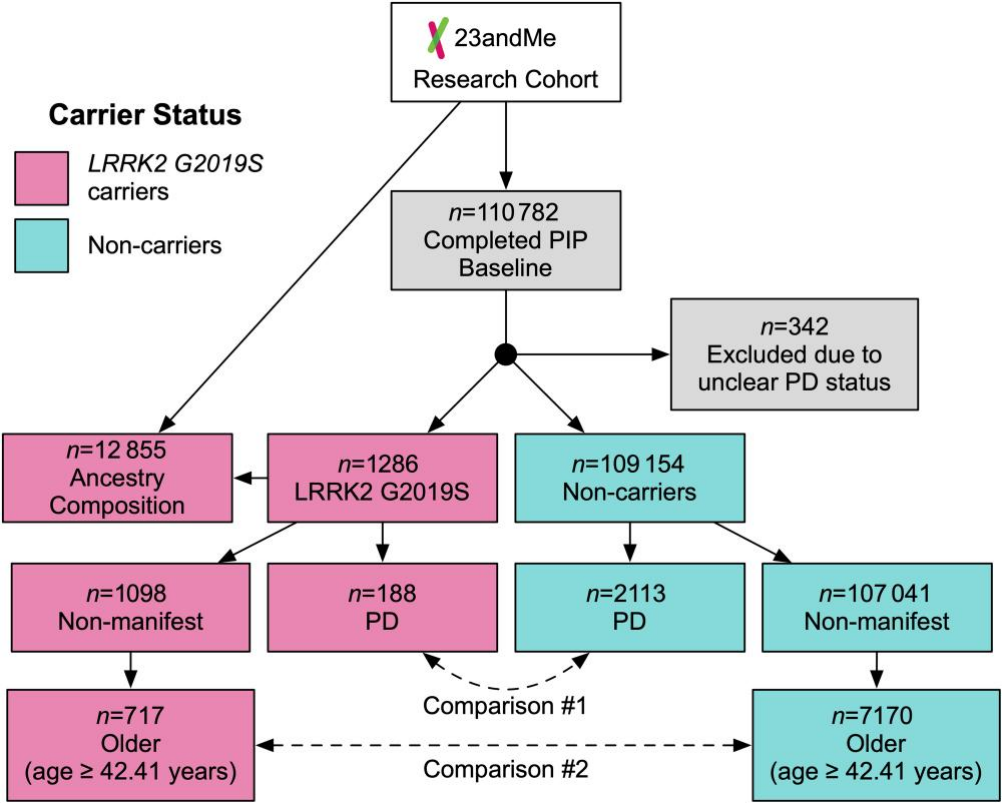
**Parkinson’s impact project study design and analysis plan.** Flow chart that shows recruitment of consented participants, classification according to *LRRK2 G2019S* carrier status and Parkinson’s disease (PD) diagnosis. The older non-manifest subgroup was selected based on age at risk of PD (≥42.41 years, i.e. within 2 SD of age of PD diagnosis for *LRRK2 G2019S* carriers). *LRRK2 G2019S* carriers received a similar survey every 6 months. Non-carriers were asked to report any new diagnosis of PD every 12 months. Pink = *LRRK2 G2019S* carriers; blue = non-carriers.

Participants were categorized according to their *LRRK2 G2019S* status (i.e. carrier versus non-carrier) and PD diagnosis (i.e. manifest versus non-manifest) creating four comparison groups: (i) *LRRK2 G2019S* PD (manifest carriers); (ii) *LRRK2 G2019S* non-manifest (*LRRK2 G2019S* carriers without PD); (iii) non-carrier PD (manifest PD without the variant); and (iv) non-carrier controls (non-carriers without PD). Given the small number of homozygous carriers (*n* < 5) and the autosomal dominant nature of *LRRK2 G2019S*, we collapsed heterozygous and homozygous carriers into one carrier group. To account for age as a risk factor for PD, we created two subgroups of non-manifest participants (Fig. 1). First, we selected a subgroup of older non-manifest *LRRK2 G2019S* carriers that had an age ≥ 42.41 years. This lower-bound age cut-off was determined by calculating 2 standard deviations (SD) below the mean age of PD diagnosis for carriers. Next, we performed 10:1 age/sex matching using a nearest-neighbour propensity score algorithm^25^ to select a comparison group of older non-carrier controls matched to the older non-manifest *LRRK2 G2019S* carriers (Fig. 1). The main statistical comparisons were: (i) *LRRK2 G2019S* PD versus non-carrier PD; and (ii) older *LRRK2 G2019S* non-manifest carriers versus older non-carrier controls. Within each comparison, we performed logistic regressions to model the effects of *LRRK2 G2019S* status. *P*-values were corrected for multiple comparisons using false discovery rate^26^ (FDR; α = 0.05) within each category (i.e. demographics, motor symptoms, prodromal markers, risk factors). Using the same matching procedure, we created a third group of non-carrier controls age- and sex-matched to all PD cases (i.e. both *LRRK2 G2019S* carriers and non-carriers) for descriptive purposes. To prevent re-identification of the data and protect participant privacy, counts with fewer than five individual participants were not reported and quoted throughout the manuscript as ‘<5’.^27^

#### Ancestry composition

To understand the ancestral origins and genetic structure of *LRRK2 G2019S* carriers, we performed a suite of ancestry analyses on all research participants who carry the *G2019S* variant. We determined the frequency of carrier prevalence around the world by comparing all research participants with *LRRK2 G2019S* in the 23andMe database (*n* = 12 855) to research participants who reported their grandparent birth locations (*n* = 545 187 outside of the USA; *n* = 2 162 527 inside the USA). Because a subset of research participants reported grandparent birth locations at subnational levels, we also performed kernel density estimation to determine subnation geographic regions that have high densities of carriers. We identified segments of DNA that are identical by descent (IBD) between all individuals using templated Positional Burrows–Wheeler Transform (TPBWT) IBD detection.^28^ TPBWT is robust to genotype and phasing errors and its default parameters are optimized to maximize accuracy on the custom designed Illumina BeadChip genotyping array. Consequently, we ran TPBWT with default parameters, but only retained IBD segments sizes >5 cM to minimize potential false-positive estimates.^28^ We identified genetic clusters of *LRRK2 G2019S* carriers using Leiden community detection on the total amount of shared IBD between individuals. We first pruned close relatives in the dataset (individuals that share ≥700 cM of IBD) to avoid producing clusters that correspond to close familial relationships. The Leiden algorithm determines clusters of individuals who share a recent common ancestor by maximizing modularity, or within-group IBD sharing related to between-group IBD sharing.^29^ We labelled any identified genetic clusters using enrichment of geographic birth locations of members’ grandparents. Finally, we employed 23andMe’s ancestry composition algorithm to infer local ancestry in genomic windows across each individual’s chromosome. Ancestry composition predicts the genomic origin of 45 local populations that are nested within broader geographic categories and is optimized for high coverage genotype array data.^30^

#### Neuroanatomical modelling of Parkinson’s disease

Survey items were grouped across six domains (cognitive, memory, autonomic, motor, smell and RBD) and differences in symptom prevalence were compared in *LRRK2 G2019S* PD versus non-carrier PD using logistic regressions accounting for disease duration, age, sex and education as covariates. Logistic regressions without covariates were separately computed using weights from coarsened exact matching^31^ to confirm results. FDR correction was applied to each model term. Further, we built an anatomical-based model of neuropathology by associating each of the six symptom domains with structural areas of the brain using the Braak framework^32^ to create a spatial representation of pathological deficits. According to the frequency of neurological symptoms reported, we colour-coded according to aggregated symptom burden in the substantia nigra (motor), cortex (cognition/memory), subarea of the pons (RBD),^33^ brainstem/periphery (autonomic), and olfactory bulb (hyposmia) to visually model patterns of neurodegeneration and recapitulate the neurobiology of PD.

#### Disease-free survival analysis

We used Kaplan-Meier estimation to describe time to PD diagnosis survival curves (i.e. using self-reported age of PD diagnosis) between *LRRK2 G2019S* carriers and non-carriers. To include prodromal risk factors, we estimated an accelerated failure time (AFT) model with a Weibull hazard shape. In addition to *LRRK2 G2019S* status, we included the following known PD risk factors as binary variables: occupational toxin exposure, male sex, caffeine non-user, non-smoker, and previous head injury with loss of consciousness. We included education (≥ associate degree) as a binary covariate. The development of PD was incident to the participants’ genetic exposure to *LRRK2 G2019S*. Therefore, participants with pre-existing PD were included in survival analyses using their self-reported age of diagnosis as the time to event. This approach is supported by recent genetic epidemiology studies.^34,35^ Participants without a PD diagnosis after 42 months or those lost to follow-up, were right censored and age at their last completed survey was used as the time of censoring. We used the fitted AFT model to calculate predicted cumulative incidence of PD across the lifespan at average risk factor and covariate values stratified by *LRRK2 G2019S* carrier status. Phenoconversion rates were estimated in participants who were ≥40 years of age at the time of study entry, as this age group is considered to be at risk. Note that the ≥40 years of age cut-off, rather than the previous 2 SD cut-off of 42.41 years, was used to calculate phenoconversion rates and was used in the PRS models to simplify useability in the clinic.

#### Polygenic risk score analysis

We computed a PRS for each participant using allelic weights from the most recently published genome-wide association studies (GWAS) meta-analysis of PD (*n* = 37.6 k PD cases, *n* = 1.4 million controls, *n* = 18.6 k proxy cases).^36^ 23andMe contributed GWAS summary statistics for *n* = 2.4 k cases (6.5% of the total cases in the GWAS) and *n* = 571.4 k controls (41%) to this meta-analysis.^37^ The overlap between 23andMe research participants used in the recent GWAS used to derive the PRS and the PIP cohort was small: 13% *LRRK2 G2019S* non-manifest, 9% *LRRK2 G2019S* PD, 6% non-carrier PD and 7% non-carrier controls; hence, overlapping participants were not removed from the present analyses.

To synthesize a PRS that could be evaluated in both carriers and non-carriers of *LRRK2* variants, we took the original PRS (1805 variants) and removed single nucleotide polymorphisms (SNPs) ±10 Mb surrounding the *LRRK2* gene, which included 46 proximal variants, to compute a modified PRS (see the Supplementary material for details). Both the original and modified PRS were scaled to have a mean of 0 and an SD of 1 (i.e. *Z*-score) using the entire PIP cohort.

We computed two logistic regressions (i.e. one for each PRS) separately for *LRRK2 G2019S* carriers and non-carriers predicting PD status as a function of PRS with baseline age, sex and 10 principal components (PCs) of ancestry as covariates (Supplementary material). We visually inspected the PCs and determined that the first 10 PCs demonstrated separation and structure for use as covariates in logistic regressions (Supplementary Fig. 1).

To examine the relationship between the PRS and risk of PD, we excluded participants < 40 years of age and split the *LRRK2 G2019S* carriers and non-carriers into six groups depending on the modified PRS percentile ranges: low (1%–25%), intermediate (25%–75%), and high (75%–100%; Fahed *et al*.^38^). We used logistic regression to model PD status as a function of PRS group with baseline age, sex and ancestry PCs as covariates. Non-carriers with intermediate PRS value served as the reference group. To examine the impact of polygenic versus monogenic risk in greater detail, we explored the association with PD risk across deciles of the modified PRS, among *LRRK2 G2019S* carriers and non-carriers, adjusting for baseline age, sex and ancestry PCs. Predicted odds ratios across carrier status were estimated using non-carriers with median (fifth decile) PRS as a reference group and mean values of the remaining covariates. To test deviations from additivity, we compared deciles models with and without the *LRRK2 G2019S* carrier status by PRS interaction term using ANOVA. PRS analyses used both baseline and incident cases of PD. Given that the Nalls *et al*.^36^ allelic weights were derived from a European-centric GWAS, all PRS analyses were repeated excluding non-Europeans to compare in our heterogeneous, but majority European, sample. The European-only analyses were identical except that only five ancestry PCs were used as covariates in addition to age and sex (Supplementary Fig. 2).

In addition, we regressed participants’ self-reported age of PD diagnosis on the modified PRS, *LRRK2 G2019S* carrier status, and their interaction in *LRRK2 G2019S* carriers and non-carriers. Participants’ sex and first 10 ancestry PCs were entered as covariates. In a second model restricted to only *LRRK2 G2019S* carriers, we regressed age of PD diagnosis on the modified PRS. Sex and the first five ancestry PCs were entered as covariates. The modified PRS and PCs were *Z*-scored across the entire PIP cohort and centred for each model. *LRRK2 G2019S* status (carriers +0.5; non-carriers −0.5) and sex (male +0.5; female −0.5) were effects coded contrasts. Incident cases were included in analyses.

#### Software and statistical analysis

Data analysis and visualization was performed using R^39^ (v. 3.6). Case-control matching was performed using *MatchIt*^25^ and verified with *cem.*^31^ Survival analyses were performed using *survival*^40^ and *flexsurv.*^41^ Figures were prepared using *ggplot2.*^42^

## Results

### Baseline characteristics

Table 1 summarizes the participant characteristics at study entry (see Supplementary Table 2 for additional groups). Figure 1 describes the participant flow and sample sizes of the comparison groups.

**Table 1:** Clinical characteristics of the Parkinson’s impact project cohort at study entry

Category	Measure	Parkinson’s disease			Older non-manifest		
		LRRK2 G2019S	Non-carriers	*P_FDR_*	LRRK2 G2019S	Matched Controls	*P_FDR_*
Demographics	*n*	188	2113	–	717	7170	–
	Male	45%	55%	0.016	42%	37%	0.017
	Age, years	68.25 (0.61)	68.95 (0.19)	0.297	61.74 (0.38)	61.94 (0.12)	0.613
	Age at PD diagnosis	60.33 (0.67)	62.25 (0.22)	0.016	–	–	–
	Disease duration	7.88 (0.45)	6.82 (0.12)	0.016	–	–	–
	BMI	26.12 (0.35)	26.95 (0.12)	0.059	27.84 (0.24)	28.61 (0.07)	0.002
	European	89%	92%	0.297	83%	87%	0.010
	Ashkenazi	61%	5%	2.19 × 10^−77^	49%	2%	2.02 × 10^−253^
	GBA N370S	6%	1%	4.12 × 10^−5^	3%	1%	1.66 × 10^−10^
	Education	83%	74%	0.016	83%	67%	5.94 × 10^−18^
Motor	Tremor	78%	81%	0.681	11%	10%	0.463
	Imbalance	68%	66%	0.715	19%	22%	0.192
	Bradykinesia	61%	66%	0.349	10%	11%	0.463
	Reduced arm swing	79%	81%	0.715	5%	6%	0.407
Prodromal markers	RBD	9%	23%	2.35 × 10^−4^	4%	2%	0.134
	MCI diagnosis	3%	8%	0.034	2%	1%	0.697
	Depression (PHQ-9^a^)	6.06 (0.37)	6.7 (0.12)	0.201	3.72 (0.15)	3.65 (0.05)	0.747
	Olfactory difficulties	32%	49%	5.73 × 10^−5^	6%	7%	0.747
	OH	36%	44%	0.085	24%	23%	0.697
	Constipation	9%	12%	0.294	5%	5%	0.883
	Urinary difficulties	51%	53%	0.600	24%	21%	0.346
	Erectile dysfunction	46%	53%	0.294	23%	26%	0.697
	Daytime somnolence	22%	24%	0.559	10%	11%	0.697
Risk factors	Occupational toxins	3%	11%	0.006	2%	5%	0.001
	Occupational PPE	28%	31%	0.685	24%	34%	3.47 × 10^−6^
	Non-smoking	62%	60%	0.722	58%	56%	0.339
	Non-use of caffeine	33%	35%	0.685	22%	27%	0.027
	Physical inactivity	6%	8%	0.685	7%	8%	0.155
	First degree relative with PD	38%	13%	6.57 × 10^−17^	32%	7%	3.92 × 10^−84^
	Head injury (with LOC)	15%	16%	0.685	11%	13%	0.075

The ages of older *LRRK2 G2019S* non-manifest participants were greater than 2 SD below the mean age of diagnosis for *LRRK2 G2019S* PD participants at baseline; continuous measures are presented as mean (standard error of the mean, SEM). BMI = body mass index; FDR = false discovery rate; LOC = loss of consciousness; MCI = mild cognitive impairment; OH = orthostatic hypotension; PD = Parkinson’s disease; PHQ-9 = Patient Health Questionnaire-9; PPE = personal protective equipment; RBD = REM sleep behaviour disorder.

^a^PHQ-9 was administered without Question 9.

#### Ancestry of LRRK2

**Figure 2:**
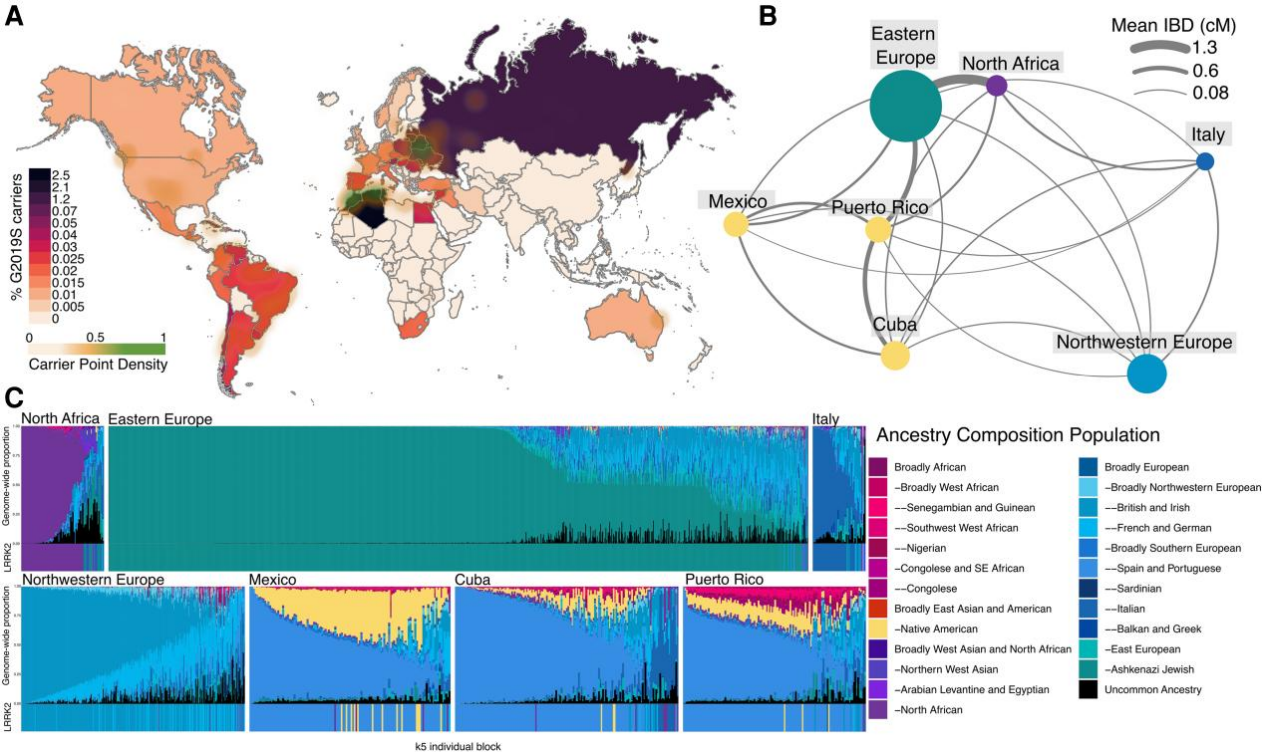
**Ancestry of *LRRK2 G2019S* carriers**. Ancestry and geographic origins of individuals who carry the *LRRK2 G2019S* mutation. (**A**) Frequency of *LRRK2 G2019S* carriers based on participant-reported grandparent birth countries. Green clouds correspond to subnational locations with the highest frequency of carriers based on kernel density estimates. (**B**) The genetic structure of the seven groups identified in *LRRK2 G2019S* carriers. Groups are arranged in a graph using ForceAtlas2 orientation based on the mean pairwise identical by descent (IBD) sharing rate. (**C**) Ancestry composition of carriers belonging to each genetic group. The mean genome-wide ancestry of individuals is displayed above, with the ancestry at *LRRK2* reflected below.

Geographical analyses in Fig. 2A showed that most *LRRK2 G2019S* carriers had grandparents born around the southern coastal regions of the Mediterranean basin, with the strongest signals in Maghreb, including Algeria (2.5% prevalence), Morocco (2.1%) and Tunisia (1.7%); eastern Europe, including modern-day Ukraine (0.7%) and Belarus (1.2%); Latin America, including Puerto Rico (0.5%), Cuba (0.6%), Colombia (0.3%) and Mexico (0.1%) (Supplementary Table 3). Leiden community detection on shared IBD segments identified seven genetic groups in *G2019S* carriers (modularity = 0.525) that correspond to geography and genetic ancestry (Supplementary Table 4). These groups included Ashkenazim from Eastern Europe (*n* = 6918), North Africa (*n* = 399), Italy (*n* = 265), Northwestern Europe (*n* = 1926), Puerto Rico (*n* = 695), Cuba (*n* = 877) and Mexico (*n* = 646). The remaining 1129 individuals were assigned to clusters with sample sizes ≤ 20 individuals and did not contain enough metadata to label. By arranging genetic groups by the mean amount of IBD shared, Ashkenazim from Eastern Europe were most genetically similar to North Africa (Fig. 2B). Puerto Rico, Mexico and Cuba groups clustered together but were most genetically similar to Eastern Europe, suggesting founder effects from migrations out of Europe into the Americas. Northwestern Europe and Italy were the most distantly connected, suggesting the mutation entered these regions through more distant migrations. Within genetic groups, ancestry inference indicated a higher proportion of Ashkenazi genetic ancestry in the Eastern Europe group, North African genetic ancestry in the North Africa group, and Iberian genetic ancestry in the Latin American groups (Fig. 2C). However, North African and Ashkenazi ancestry was present across individuals within the Latin American groups (Supplementary Fig. 3).

#### Parkinson’s disease: LRRK2 G2019S carriers versus non-carriers

**Figure 3:**
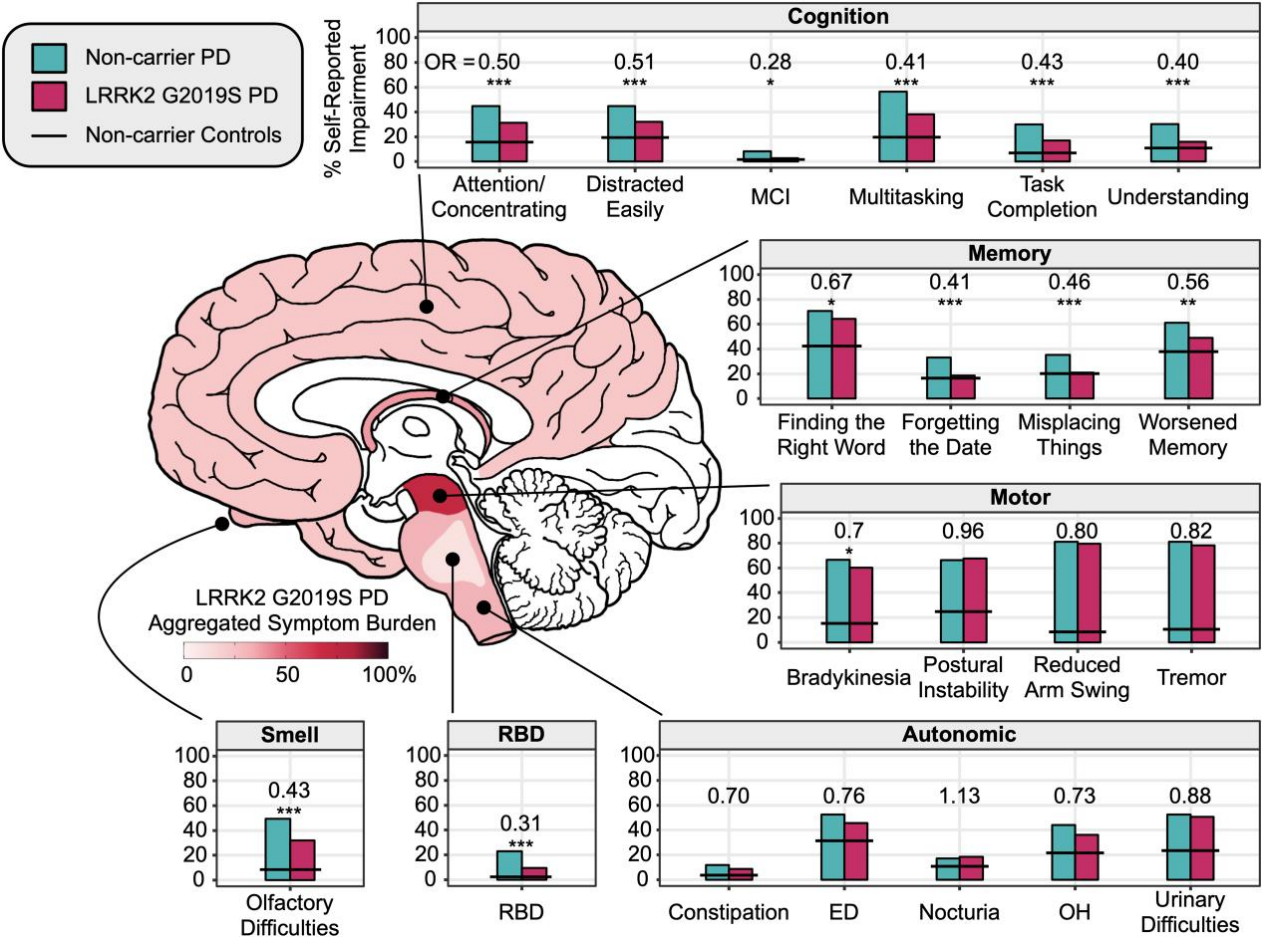
**Relative to non-carrier PD, *LRRK2 G2019S* PD reported lower symptomatic burden in cognitive, memory, autonomic, RBD and olfactory domains**. Symptoms were clustered by domain. Bars show the percentage of *LRRK2 G2019S* Parkinson’s disease (PD) carriers and non-carrier PD that self-reported a symptom. Differences between groups were estimated using odds ratios (OR) from logistic regressions adjusted for age, sex, education and disease duration. Horizontal black lines show prevalence reported by age- and sex-matched non-carrier controls (not included in modelling). Schematic of the brain shows corresponding neuropathological sites underlying symptom burden for *LRRK2 G2019S* PD. Aggregated symptom burden was calculated by averaging the percentage of reported symptoms across each phenotype domain in *LRRK2 G2019S* PD. Note the similarities in the reported frequency of motor symptoms, but lower prevalence for symptoms corresponding to regions outside the basal ganglia (e.g. RBD, smell, cognition). *P < 0.05; **P < 0.01; ***P < 0.001. ED = erectile dysfunction; MCI = mild cognitive impairment diagnosis; OH = orthostatic hypotension; OR = odds ratio comparing *LRRK2 G2019S* PD and non-carrier PD; RBD = REM sleep behaviour disorder. The brain illustration and colour shading was reprinted with modifications from Braak *et al*.^43^ with permission from Elsevier.

At entry, *n* = 188 *LRRK2 G2019S* carriers and *n* = 2113 non-carriers reported a diagnosis of PD (Fig. 1). Relative to non-carriers, the *LRRK2 G2019S* carriers were more likely to be female, have a higher level of education, be of Ashkenazi Jewish descent, and have a first degree relative with PD (Table 1). *LRRK2 G2019S* carriers were also more likely to carry the *GBA N370S* variant. Lifestyle risk factors were similar between the groups (i.e. non-smoking, non-use of caffeine, head injuries and physical inactivity). Despite being of similar age at study entry, *LRRK2 G2019S* carriers reported their age of PD diagnosis 1.9 years younger [standard error (SE) = 0.7 years; *P* = 0.027] than non-carriers, resulting in a longer disease duration at the time of entry. Figure 3 shows differences between symptom prevalence in *LRRK2 G2019S* PD and non-carrier PD (Supplementary Table 5). *LRRK2 G2019S* PD participants had a similar burden of motor symptoms to PD non-carriers yet reported significantly fewer non-motor symptoms. Compared to non-carrier PD, *LRRK2 G2019S* PD reported lower rates of RBD (23% versus 9%), lower rates of olfactory deficits (49% versus 32%) and fewer MCI diagnoses (8% versus 3%). No differences in autonomic symptoms were observed. Modelling of symptom domains with underlying anatomical mapping showed that the motor regions were equally impacted, but suggested less pathology outside the substantia nigra in *LRRK2 G2019S* PD compared to non-carrier PD.

### Phenoconversions to Parkinson’s disease

In participants without a PD diagnosis at entry, there were no differences in the rates of reported symptoms in older *LRRK2 G2019S* carriers (*n* = 717) and matched older non-carrier controls (*n* = 7170). Over the 3.5 years of follow-up in asymptomatic participants ≥ 40 years of age, we observed five newly diagnosed cases of PD in *LRRK2 G2019S* carriers and 53 new cases in non-carriers. Thus, prospectively *LRRK2 G2019S* carriers had an estimated 10-times the risk of developing PD compared to non-carrier controls (0.19 versus 0.019%/year). Put differently, we observed five incident cases of PD per 1000 person-years in *LRRK2 G2019S* carriers. *LRRK2 G2019S* carriers that developed PD were on average 6.6 years (SE = 2.0 years) younger in age at PD diagnosis compared to non-carriers newly diagnosed with PD. Prior to diagnosis, the most commonly reported non-motor symptoms reported by the non-carriers that subsequently developed PD included RBD diagnosis/dream re-enactment behaviour (13%) and smell loss (27%). In contrast, none of the five *LRRK2 G2019S* cases reported suspected RBD prior to their PD diagnosis. Prodromal autonomic symptoms reported in non-carriers prior to diagnosis included erectile dysfunction in males (48%), urinary difficulties (43%) and orthostatic hypotension (37%). There was no clear pattern of prodromal autonomic symptoms in *LRRK2 G2019S* carriers prior to phenoconversion.

### Parkinson’s disease-free survival

**Figure 4:**
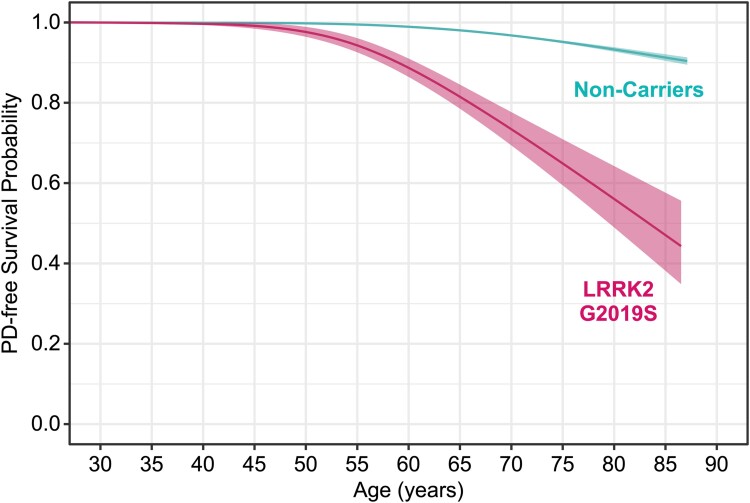
**Survival curves with Kaplan-Meier estimation show a steeper decline in PD-free survival in *LRRK2 G2019S* carriers (*n* = 1154) relative to non-carriers (*n* = 97 308)**. Curves were smoothed using generalized additive models to protect data privacy. Shading depicts 95% confidence intervals. PD = Parkinson’s disease.

Figure 4 shows PD-free survival curves with Kaplan-Meier estimation of the cohort stratified according to their *LRRK2 G2019S* carrier status. We computed a Weibull AFT model that included known prodromal risk factors of PD on a subset of participants who had non-missing data across all covariates: *n* = 772 *LRRK2 G2019S* non-manifest, 162 *LRRK2 G2019S* PD, 1574 non-carrier PD and 78 445 non-carrier controls. The AFT model showed that *LRRK2 G2019S* carrier status had the greatest impact on the age of PD diagnosis (Table 2). The estimated coefficient for carriers was −0.32 95% confidence interval (CI) (−0.35, −0.30), suggesting an accelerated rate of receiving a PD diagnosis. Correspondingly, the time ratio of 0.72 indicates that, on average, *LRRK2 G2019S* carriers were diagnosed with PD ∼28% earlier compared to non-carriers. Other known PD risk factors had detectable, but comparably modest effects on PD-free survival rates (3%–11%). The predicted cumulative incidence of PD in *LRRK2 G2019S* carriers was much greater than non-carriers at average risk factor exposure and education, despite having similar increases per decade from 40 to 80 years of age (Table 3).

**Table 2:** Weibull accelerated failure time model coefficients and time ratios of Parkinson’s disease-free survival

Term	Coefficient	95% CI [LL UL]	SE	W	Time ratio	95% CI	*P*
LRRK2 G2019S	−0.32	[−0.35, −0.3]	0.013	−24.72	0.72	[0.71, 0.74]	7.02 × 10^−135^
Occupational toxin exposure	−0.11	[−0.13, −0.09]	0.012	−9.61	0.89	[0.87, 0.91]	7.30 × 10^−22^
Male	−0.06	[−0.07, −0.05]	0.007	−8.30	0.94	[0.93, 0.96]	1.02 × 10^−16^
Caffeine non-use	−0.06	[−0.07, −0.04]	0.007	−7.76	0.94	[0.93, 0.96]	8.76 × 10^−15^
Non-smoking	−0.05	[−0.07, −0.04]	0.007	−7.21	0.95	[0.94, 0.96]	5.64 × 10^−13^
Previous head injury with LOC	−0.04	[−0.05, −0.02]	0.009	−3.74	0.97	[0.95, 0.98]	1.85 × 10^−4^
Education (≥ associate degree)	−0.03	[−0.04, −0.01]	0.008	−3.22	0.97	[0.96, 0.99]	1.26 × 10^−3^
Shape	6.97	[6.73, 7.22]	0.126	–	–	–	–
Scale	128.90	[125.44, 132.46]	1.790	–	–	–	–

CI = confidence interval; LL = lower level; LOC = loss of consciousness; UL = upper level; SE = standard error; W = Wald test statistic.

**Table 3:** Predicted cumulative incidence of Parkinson’s disease for *LRRK2 G2019S* carriers and non-carriers

Age (years)	LRRK2 G2019S			Non-carriers		
	Cumulative incidence (%)	95% CI		Cumulative incidence (%)	95% CI	
		LL	UL		LL	UL
40	0.54	0.44	0.65	0.06	0.05	0.07
50	2.51	2.13	2.98	0.27	0.24	0.30
60	8.66	7.42	10.02	0.95	0.88	1.02
70	23.30	20.29	26.41	2.74	2.61	2.90
80	48.97	43.61	54.32	6.81	6.45	7.24

Cumulative incidence was calculated from observed Weibull accelerated failure time (AFT) model estimates across average risk factor and covariate values. CI = confidence interval; LL = lower level; UL = upper level.

### Polygenic risk scores

**Figure 5:**
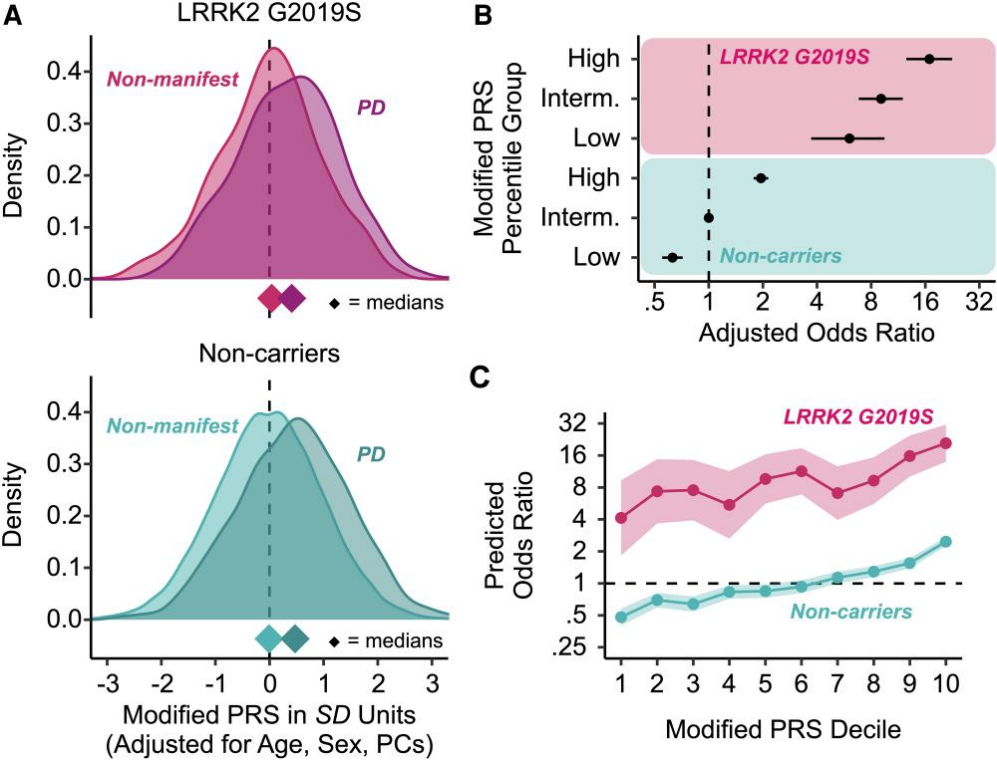
**Associations between PRS and PD prevalence for participants ≥ 40 years of age.** (**A**) Distributions of the modified polygenic risk scores (PRS) adjusted for age, sex and ancestry principal components (PCs). Diamonds indicate median PRS. Non-manifest non-carriers were the non-carrier controls. (**B**) Odds ratios of Parkinson’s disease (PD) diagnosis between *LRRK2 G2019S* carriers and non-carriers stratified by PRS percentile groups: low (0%–25%), intermediate (25%–75%), high (75%–100%). Logistic regressions were adjusted for age, sex and ancestry PCs, and non-carriers with intermediate PRS were the reference group. Error bars are 95% confidence intervals (CIs). (**C**) Predicted PD odds ratios between *LRRK2 G2019S* carriers and non-carriers referenced to non-carriers with median PRS across PRS deciles. Shading denotes 95% CIs. Interm. = intermediate; SD = standard deviation.

We identified similar odds ratios for associations between PD and both the original and modified PRS that excluded the *LRRK2* gene (Supplementary Table 6; European-only results in Supplementary Table 7). To explore differences in polygenicity to PD risk among *LRRK2 G2019S* carriers and non-carriers, we compared the distributions adjusted for age, sex and ancestry PCs of the modified PRS between individuals with and without PD. PD cases had a greater median PRS compared to those without a PD diagnosis for both carriers and non-carriers of *LRRK2 G2019S* (Fig. 5A). *LRRK2 G2019S* carriers with and without PD reported similar frequencies of risk factors, while non-carriers with PD reported more risk factors than non-carrier controls (Supplementary Table 8).

We examined dose-response relationships between the modified PRS and PD using logistic regression. Non-carriers at the median of the PRS served as the reference group. Both the group-wise (i.e. low, middle, high PRS) and decile models demonstrated a positive monotonic relationship between PRS and relative odds of PD (Supplementary Table 9; see Supplementary Table 10 for European-only results). Compared to non-carriers in the middle of the PRS distribution (i.e. intermediate), non-carriers in the top 25% of the PRS had nearly double the relative odds of PD [odds ratio (OR) = 1.95 (1.78 2.14), *P* = 2.03 × 10^−44^] and non-carriers in bottom 25% PRS had a lower relative odds of PD [OR = 0.63 (0.55 0.72), *P* = 4.08 × 10^−12^]. Using the same reference group, the relative odds of PD among *LRRK2 G2019S* carriers was substantially greater than non-carriers. Compared to intermediate non-carriers, among *LRRK2 G2019S* carriers in the bottom 25% of the PRS, the relative odds was 6-fold greater [OR = 6.06 (3.71 9.49), *P* = 4.24 × 10^−14^], for those in the middle of the PRS it was 9-fold greater [OR = 9.09 (6.82 11.99), *P* = 2.94 × 10^−53^] and among those in the top 25% of the PRS it was 16-fold greater [OR = 16.87 (12.55 22.55), *P* = 7.73 × 10^−80^] (Fig. 5B). Taking a more extreme comparison, *LRRK2 G2019S* carriers in the top 25% of the PRS had a 27-times greater relative odds of PD compared to non-carriers in the bottom 25% of the PRS.

Examining these associations in greater detail using deciles of the modified PRS relative to non-carriers at the fifth PRS decile (median) yielded predicted odds ratios that were between six-times (seventh decile) and 12-times (sixth decile) greater across all PRS deciles among *LRRK2 G2019S* carriers compared to non-carriers (Fig. 5C). For example, relative to non-carriers in the fifth PRS decile, the predicted odds ratio among non-carriers in the top decile was 2.92 compared to an odds ratio of 24.69 among *LRRK2 G2019S* carriers in the same decile (Supplementary Table 11; see Supplementary Table 12 for European-only results). An ANOVA comparing the additive model, which includes PRS decile and *LRRK2 G2019S* status as separate terms, to the interactive model, which includes their interaction, demonstrated that deviations from additivity were not observed in the decile model (*P* = 0.71). Removing *GBA N370S* carriers did not change the findings. Taken together, these findings demonstrate the additive effect of LRRK2 on top of underlying polygenic risk of PD and highlights that among *LRRK2 G2019S* carriers, polygenicity explains some of the variability in whether individuals develop PD. Similar results were obtained for participants with European-only ancestry.

A higher PRS was associated with an earlier age of PD diagnosis. In *LRRK2 G2019S* carriers, for each SD increase in the modified PRS, we observed a 1.35 year (0.02 2.67) earlier age of PD diagnosis (*P* = 0.0467). We observed a similar association between a greater PRS and earlier age of diagnosis in the entire cohort, but of a lower magnitude [1.25 years (0.52 1.98) per SD increase in the modified PRS, *P* = 8.13 × 10^−4^] (Supplementary Table 13).

## Discussion

In this study involving 1286 *LRRK2 G2019S* carriers corresponding to 1621 person-years of follow-up (1292 person-years at risk of PD) and 109 154 non-carriers, we show that the predicted cumulative incidence of PD in *LRRK2 G2019S* carriers by age 60 is 9% and 49% by age 80 years. One novel finding of our study is the discovery that the penetrance of *LRRK2 G2019S* is influenced by polygenicity, with the risk of a PD diagnosis increasing 16-fold in carriers that also have a PRS within the top 25th percentile relative to non-carriers in the middle range of the PRS (i.e. 25th–75th percentile). However, the *LRRK2 G2019S* variant still carries a risk above and beyond polygenicity, with carriers at lowest polygenic risk having greater relative odds of PD than non-carriers at the highest polygenic risk (Fig. 5).

The clinical expression of *LRRK2 G2019S* PD appears to be different than in non-carriers with idiopathic PD. Overall, we found that *LRRK2 G2019S* carriers with PD were less likely to report non-motor features, such as olfactory deficits and RBD. Moreover, cognitive symptoms were consistently reported at a lower prevalence in *LRRK2 G2019S* carriers with PD compared to non-carriers with idiopathic PD, which aligns with cross-sectional studies using cognitive function tests.^44,45^ We showed that despite a longer disease duration, *LRRK2 G2019S* PD was associated with a similar prevalence of motor symptoms, including postural instability, suggesting a slower rate of progression. Taken together, our findings support the concept that the *LRRK2 G2019S* variant produces a milder form of parkinsonism with less impact to regions outside the substantia nigra (Fig. 3), and aligns with observations in clinical cohorts.^13,46^ The differences in the clinical expression of PD in *LRRK2 G2019S* carriers compared to non-carriers with idiopathic PD may reflect differences in the core biology of *LRRK2 G2019S* PD, with α-synuclein aggregation in the CSF and Lewy body pathology at autopsy being absent in one-third of *LRRK2* carriers with PD.^21^ This may be especially relevant as Lewy body pathology has been shown to have independent negative effects on global cognition and memory in longitudinal studies.^47^

Our findings have important implications for prodromal clinical trial designs in *LRRK2 G2019S* carriers. First, our results show that a high PRS confers an additional risk of PD in *LRRK2 G2019S* carriers and earlier reported PD diagnoses, suggesting that PRS may be useful as an enrichment stratification biomarker for candidate optimization^48,49^ and symptom progression.^50,51^ Likewise, previous studies have similarly demonstrated relationships between PRS and PD status^52^ and age at onset^52-55^ further supporting the utility of PRS in PD clinical trials. Second, we show that although not always absent, RBD and olfactory deficits are less prevalent in *LRRK2 G2019S* PD. This finding suggests that the current prodromal criteria that rely heavily on these non-motor features to predict the future likelihood of PD, may under-estimate the risk in *LRRK2 G2019S* carriers. Third, our phenoconversion rates to PD in *LRRK2 G2019S* carriers, although 10-fold higher than non-carriers, indicate large sample sizes would still be needed to adequately power clinical trials.^11^ This suggests that phenoconversion is unlikely to be a good primary outcome measure until we find a way to enrich the candidate section. Finally, we did not detect an increased prevalence of self-reported motor symptoms in older *LRRK2 G2019S* non-manifest carriers compared to matched non-carrier controls. Together, these findings underscore the need for objective end points that are sensitive to milder parkinsonian symptoms in the earlier phases of neurodegeneration in high-risk cohorts.

The geographic distribution of present-day *LRRK2 G2019S* carriers both aligns with the known history surrounding the dispersion of the variant while also revealing novel insights regarding the population structure.^3,56^ High carrier rates in North Africa support the concept of an ancient Moroccan Berber founder. The enriched geographic location in Eastern Europe—specifically in the area corresponding to the Pale of Settlement—aligns with the hypothesis of a subsequent flow of the *LRRK2 G2019S* variant into the Ashkenazi Jewish community, likely predating the 14th century bottleneck.^3,4,56-58^

The discovery of carrier populations in the Latin Caribbean is novel. The Spanish colonization of these islands began in 1492, with the voyages of Columbus, which would introduce Iberian genetic ancestry into native populations. While Iberian ancestry is common among Puerto Rican and Cuban *LRRK2 G2019S* carriers in our cohort, North African and Ashkenazi ancestry was also present at lower frequencies in nearly all individuals. Historical records, previous ancestry analyses and our findings align with the theory that the *LRRK2 G2019S* allele may have been introduced to the Caribbean by the male Sephardic Jewish Conversos, who originally entered Spain via North Africa^59^ and subsequently fled the Inquisitions, sailing to the new world as Conquistadors to become part of the founding populations.^60,61^ Mexican *LRRK2 G2019S* carriers, though sharing similar patterns of genetic ancestry with Latin Caribbean carriers, tended to have higher Ashkenazi ancestry with little North African ancestry. Jewish Conversos may have contributed to the introduction of the *LRRK2 G2019S* variant into Mexico, but the lack of North African ancestry suggests that the mutation may have originated primarily from the immigration of Yiddish-speaking Ashkenazim from eastern Europe following Mexico’s 19th century Liberal reform, which included toleration of non-Catholic religions.^62^ Similarly, the influx of Eastern European Ashkenazi Jews who emigrated from the Pale of Settlement to South America likely also contributed to the high *LRRK2 G2019S* carrier rates that were observed in Uruguay, Argentina and Brazil.^63^ Across Latin America, these founding populations remained geographically isolated, went through additional bottlenecks, followed by periods of population expansion, leading to a higher burden of rare variants.^64^ Ultimately, colonization, migration due to religious persecution and other diasporas introduced the *LRRK2 G2019S* allele into populations outside of Europe and North Africa.

A major advantage of our study is the sample size of 23andMe’s *LRRK2 G2019S* cohort enrolled in this prospective study, which exceeds other published cohorts by nearly 3-fold. Our findings in *LRRK2 G2019S* carriers are supported by a control group of over 2000 non-carrier idiopathic PD patients and 100 000 controls, enabling comparisons with well powered age-matched groups. The phenotypic data collection points used in the study were collected through online self-reported answers that provide a unique insight into the patient’s ‘voice’ and functional implications. These self-report measures indicated that motor symptoms, particularly tremor and postural instability, are the most prominent features of *LRRK2 G2019S* carriers with PD. These differences in sample size (i.e. especially right-censored non-manifest carriers and non-carriers), community-based recruitment and event measures (i.e. age of PD diagnosis) may explain why our observed cumulative incidence of *LRRK2 G2019S* PD was lower compared to previously published estimates.^9,65^

There are several limitations to consider. First, the cohort of *LRRK2 G2019S* carriers at 23andMe may be unique in some respects, as they are considered more affluent, educated and perhaps more concerned about their health to seek out direct-to-consumer genetic testing. This may have introduced selection bias in our risk factor assessment, which may limit the generalizability of the data when it comes to symptom reporting in populations that are less health conscious. Nevertheless, they are assembled by virtue of them choosing direct-to-consumer genetic testing and thus not biased by clinical presentation within a neurological subspecialty, as occurs in many prodromal cohorts. We relied on self-reported PD diagnosis but have shown 100% concordance (*k* = 1.00) with neurologist-confirmed diagnoses in a validation study of 23andMe’s Parkinson’s Disease Community.^66^ Moreover, we reviewed information available in other surveys, which confirmed that the participants with PD reported being diagnosed by a neurologist (95.2%) or other physician (4.1%). 23andMe has demonstrated high validity with self-reported symptom measures.^67,68^ We are aware that RBD may be under-reported in patients without a bed partner and some patients may be unaware that they have olfactory deficits, but this is unlikely to invalidate our findings as the prevalence of under-reporting is expected to be similar in carriers and non-carriers. There does, however, appear to be good concordance between self-reported motor symptoms of PD and findings on neurological examination.^69^ Compared to non-carriers with idiopathic PD, *LRRK2 G2019S* carriers with PD reported a 2-year earlier age of PD diagnosis. Given that these participants were aware they were carriers of the *G2019S* variant, it is possible that knowing their genetic predisposition may have prompted them to seek neurological care sooner and/or may have influenced physician diagnosis, thus resulting in an earlier age of diagnosis estimate. It is therefore possible that the 2-year difference in age of PD diagnosis could be overestimated because of these biases. Nevertheless, the finding highlights the potential usefulness of direct-to-consumer genetic testing in early disease detection in at-risk cohorts, which may lead to more prompt diagnosis. Additionally, loss to follow-up poses a challenge in providing robust estimates, especially of prospective phenoconversion rates, which may have been underestimated. As we saw the lowest rates of dropout in the older *LRRK2 G2019S* carriers, we focused our comparisons on participants ≥42.41 years of age, who had the better follow-up rates (Supplementary Table 1). We did not have a sufficient number of homozygous *LRRK2 G2019S* carriers (*n* < 5) or dual carriers with *GBA N370S* to draw significant conclusions about these subgroups, other than the fact they exist. Larger sample sizes are needed to determine whether the *LRRK2 G2019S* mutation offers some protection in dual carriers with a *GBA N370S* mutation, who do not appear to have an earlier onset of disease.^70^ We did not account for the carrier rate of rare *GBA* variants beyond the most common *N370S* that may have influenced the age of diagnosis in the cohort. Therefore, we are unable to conclude whether the *GBA* locus had an effect on participants’ age of PD diagnosis. Further work is needed to determine if the findings are generalizable to carriers of other *LRRK2* variants beyond *G2019S*. Our results are restricted to participants based in the USA and may not be generalizable to populations in other countries. While the high carrier rates seen in North Africa and other geographic regions may be indicative of a historical founder effect, it is possible that a combination of other evolutionary processes, such as selection or genetic drift by means of a bottleneck, could produce the same patterns seen elsewhere in the world. In future research, the influence of each process should be explicitly tested in populations with high carrier rates.

The question remains as to why one-third of *LRRK2* PD patients do not show α-synuclein pathology at autopsy^19^ and have a negative α-synuclein seeding amplification assay test.^12^ This is important to understand as anti-α-synuclein based treatment strategies could potentially lack efficacy in a subset of *LRRK2 G2019S* carriers and/or these patients may not meet eligibility criteria to enter trials and test new therapies. However, this raises the bigger unanswered question of where *LRRK2 G2019S* carriers—who meet clinical diagnostic criteria for PD but lack evidence of α-synuclein seeding—fit in the new biological definitions of PD that are likely to emerge, especially as the causal role of α-synuclein aggregates in PD is still being debated.^71,72^

In summary, this study shows that *LRRK2 G2019S* PD is associated with fewer self-reported cognitive and olfactory deficits and a lower prevalence of RBD. The findings may have implications for the design of early interventional trials in *LRRK2 G2019S* carriers, including the need for more precise prodromal clinical criteria to detect early disease onset, using high PRS scores as an enrichment biomarker to optimize candidate selection for a high yield of phenoconverters, and developing more sensitive end points for tracking this slow progressing mild motor subtype of PD. Finally, our ancestry results showing high carrier rates throughout the Latin Caribbean and South America may be helpful for selecting populations in other countries for genetic screening as ways to advance clinical trial recruitment in *LRRK2 G2019S* carriers.

## Supplementary Material

awae073_Supplementary_Data

## Data Availability

Model outputs for all logistic regressions are provided as Supplementary material. Individual-level data are not publicly available due to participant confidentiality and in accordance with the IRB-approved protocol under which the study was conducted. No custom code or software was generated as part of the study. Details of all software packages used for data processing and/or analysis may be found in the ‘Materials and methods’ section.

## Appendix 1

The following members of the 23andMe Research Team contributed to this study:

Adam Auton, Elizabeth Babalola, Robert K. Bell, Jessica Bielenberg, Johnathan Bowes, Katarzyna Bryc, Ninad S. Chaudhary, Sayantan Das, Emily DelloRusso, Sarah L. Elson, Nicholas Eriksson, Will Freyman, Julie M. Granka, Alejandro Hernandez, Barry Hicks, Ethan M. Jewett, Yunxuan Jiang, Katelyn Kukar, Alan Kwong, Keng-Han Lin, Bianca A. Llamas, Maya Lowe, Matthew H. McIntyre, Meghan E. Moreno, Priyanka Nandakumar, Dominique T. Nguyen, Jared O'Connell, Aaron A. Petrakovitz, G. David Poznik, Alexandra Reynoso, Morgan Schumacher, Leah Selcer, Anjali J. Shastri, Qiaojuan Jane Su, Susana A. Tat, Vinh Tran, Xin Wang, Wei Wang, Catherine H. Weldon, Peter Wilton, Corinna D. Wong.
