# Supplementary material for: Genetic analysis and natural history of Parkinson’s disease due to the LRRK2 G2019S variant
Source: Brain. 2024 May 28;147(6):1996–2008. doi: 10.1093/brain/awae073 (PMC11146432; doi:10.1093/brain/awae073)
Supplement: awae073_Supplementary_Data [file awae073_supplementary_data.pdf]

Supplementary Material

Genotyping

The V1 and V2 platforms were variants of the Illumina HumanHap550+ BeadChip and contained a total of approximately 560,000 SNPs, including about 25,000 custom SNPs selected by 23andMe. The V3 platform was based on the Illumina OmniExpress + BeadChip and contained a total of about 950,000 SNPs and custom content to improve the overlap with our V2 array. The V4 platform is a fully custom array and includes a lower redundancy subset of V2 and V3 SNPs with additional coverage of lower-frequency coding variation, and about 570,000 SNPs. The V5 platform is an Illumina Infinium Global Screening Array of about 640,000 SNPs supplemented with about 50,000 SNPs of custom content.

Imputation Reference Panels

Variants were imputed using a reference panel comprised of three other reference panels: the publicly available Human Reference Consortium (HRC) and UK BioBank (UKBB) 200K Whole Exome Sequencing (WES) reference panels, and the 23andMe reference panel. HRC data were downloaded from the European Genome-Phenome Archive at the European Bioinformatics Institute (accession EGAD00001002729). Variants were converted to hg38 and excluded if their new positions were on a different chromosome. Variants were then re-phased using SHAPEIT4 (https://odelaneau.github.io/shapeit4/). Finally, singletons were excluded. The final HRC reference panel included 27,165 samples and 39,057,040 SNPs (no indels).

The UKBB 200K WES reference panel was built following a recently published approach (Barton et al., 2021). UKBB genotyping data included 486,633 samples and 681,298 variants, and was lifted from hg19 to hg38 and re-phased using SHAPEIT4. UKBB 200K WES data included 200,643 whole exome sequenced samples (mostly British ancestry) and more than 22M variants (https://www.ukbiobank.ac.uk/media/cfulxh52/uk-biobank-exome-release-faq_v9-december-2020.pdf). Multi-allelic variants were split into bi-allelic variants using bcftools. Genotypes with GQ<20 were set to missing. Variants with >20% missingness, a minor allele count of 0, or an inbreeding coefficient < -0.3 (high heterozygosity) were removed. After QC, 17,975,023 UKBB WES variants remained. For the 199,815 samples with both WES and genotype array data, the variants were merged and re-phased using SHAPEIT4. After excluding singletons, the UKBB 200K WES reference panel included 199,815 samples and 17,082,588 variants (16,115,376 SNPs and 967,212 indels).

The 23andMe reference panel included 12,217 samples from: consented 23andMe customers, the 1000 Genomes Project (Auton et al., 2015), Syndip (Li et al., 2018), the Genotype-Tissue Expression (GTEx) project v8 (Aguet et al., 2019), the Human Genome Diversity Project (HGDP) (Bergström et al., 2020), and the Simons Genome Diversity Project (SGDP) (Mallick et al., 2016). All samples (except for those from GTEx v8) were consented for research as of 2020-01-18, had sufficient depth of coverage (greater than the median - 3*MAD (median absolute deviation) within each cohort), had contamination < 0.05 as estimated by *verifybamid*, had <0.05 chimeric reads, had median insert size >= 250 bp, had r2 with genotyping array >= 0.8, and had >= 3M SNPs called. GTEx v8 samples were sometimes slightly outside these bounds, but were included due to their value in eQTL mapping. All samples were aligned to GRCh38_full_analysis_set_plus_decoy_hla.fa9 and duplicate marked. Datasets sequenced before 01/01/2019 were re-aligned using an in-house pipeline consisting of bwa mem 0.7.15-r1140 alignment and duplicate marking with samblaster v0.1.24. Datasets sequenced after 01/01/2019 were processed using a well-known public pipeline from the Broad Institute (Aguet

et al., 2019) that combined bwa mem 0.7.15-r1140 alignment, Picard MarkDuplicates 2.15.0, and BQSR with GATK 4.beta.5. Variants were called in each individual sample using DeepVariant-0.8.0 11 to produce GVCFs. The GVCFs were then joint-called using GLnexus-1.2.3 12. Singletons were removed, genotypes with GQ<20 were set to missing, variants with >20% missingness (after the GQ filter) were removed, and variants with >30% excess heterozygosity were removed. Finally, variants were phased using SHAPEIT4. SHAPEIT4 also imputed missing genotypes and produced a final panel without missingness. The final 23andMe reference panel included 12,217 samples and 82,078,539 variants (73,852,355 SNPs + 8,226,184 indels).

Imputation

Imputation was performed using Beagle 5 (Browning et al., 2018). Because participants were genotyped on one of the five genotyping platforms (v1 to v5), imputation was performed independently for each platform. Similarly, participants were classified into one of five ancestry-based populations as described previously (https://www.23andme.com/ancestry-composition-guide/) and imputation was performed independently for each population. We used a two-step imputation process: first combining results from the HRC and 23andMe reference panels, then combining the HRC+23andMe results with UKBB 200K WES results. In the first step, there were 85,099,656 variants found in either the HRC or 23andMe reference panels. HRC-specific variants (n=3,021,117) were imputed using all HRC reference panel samples. 23andMe-specific variants (n=46,042,616) were imputed using all 23andMe reference panel samples. Variants found in both HRC and 23andMe reference panels (hereafter referred to as HRC+23andMe, n= 36,035,923) were imputed using all HRC+23andMe reference panel samples. In the second step, there were 99,675,338 variants found in either the HRC+23andMe or UKBB 200K WES reference panels. HRC+23andMe-specific variants were imputed as described in step 1 (n=82,592,750). UKBB 200K WES-specific variants were imputed using the UKBB 200K WES reference panel (n= 14,575,682). Variants found in both the HRC+23andMe and the UKBB 200K WES reference panels (n= 2,506,906) were imputed in each panel separately and the result with the higher imputation quality was used. Imputation quality was taken as the sample size-weighted imputation R2 value across genotyping platforms and populations.

Polygenic Risk Score Calculation

Both the original and modified PRS were a weighted sum of risk allele counts for the included SNPs that were matched using CPRA (chromosome, position, reference allele, alternative allele) format and harmonized to 23andMe imputation panel (Hartwig et al., 2016). We removed SNPs with imputation R^2<0.5 or a difference in MAF>30% between the Nalls et al (2019) and 23andMe variants. These quality control procedures removed an additional 69 SNPs from both PRS (67 unmatched and 2 low R^2 variants) resulting in a total of 1,770 and 1,724 variants for the original and modified PRS, respectively.

Principal Components of Ancestry

PCs were derived via principal component analysis performed on one million randomly selected participants from 23andMe's Research Cohort using 63,528 high quality genotyped variants present across all five genotyping platforms. Loadings for participants not included in the analysis were obtained by projection, combining the eigenvectors of the analysis and the SNP weights.

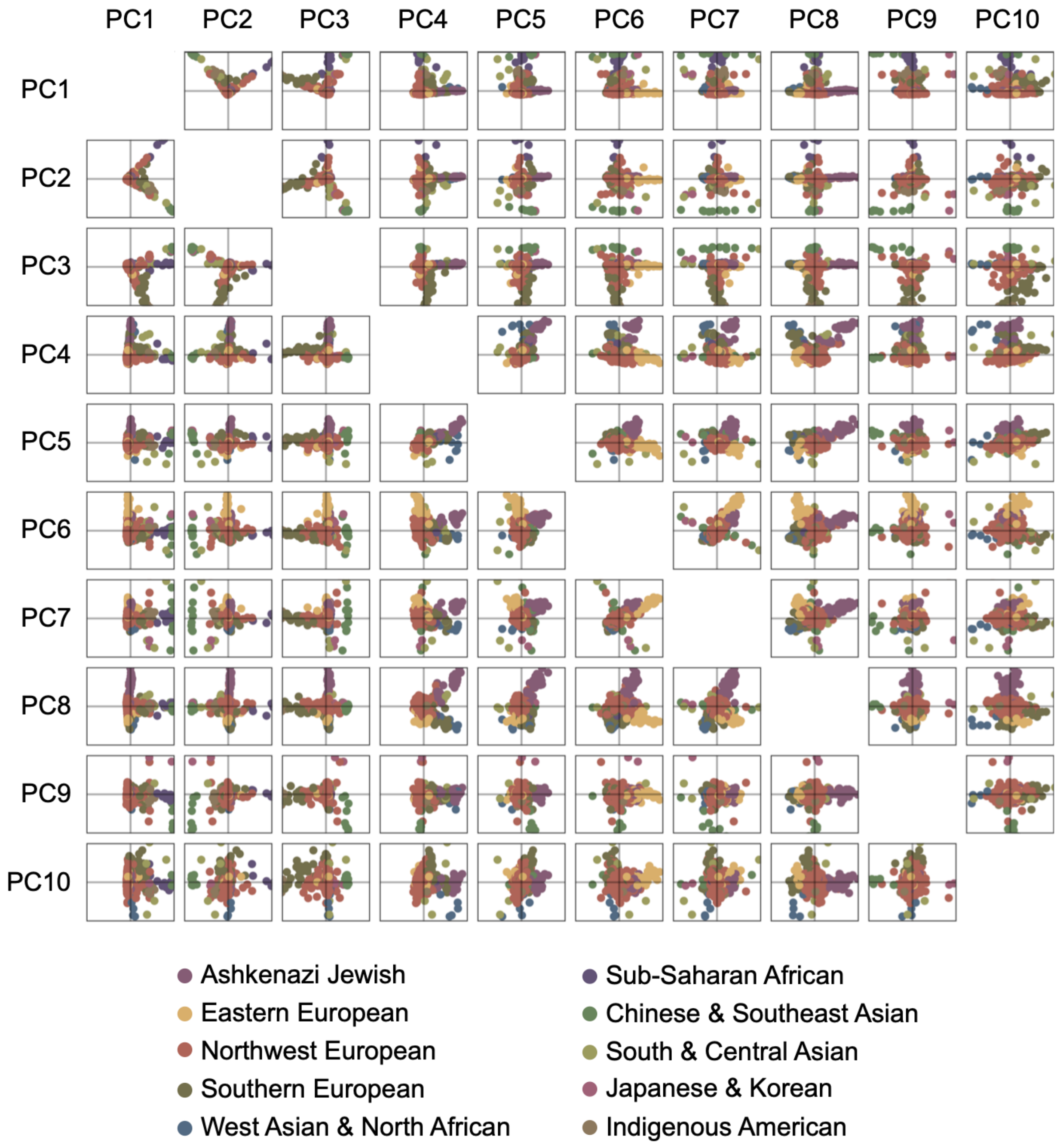

Supplementary Figure 1. Scatter matrix of the first 10 ancestry principal components (PCs) plotted from a random sampling of *n*=1,000 PIP participants. We classified individuals by their maximum genetic ancestry using 12 regional populations predicted by Ancestry Composition (Durand et al., 2014). 0.03% of participants with ancestry that is rare in the PIP cohort (i.e., Oceanian or Northern Asian) were excluded in this random sample and are therefore not visualized.

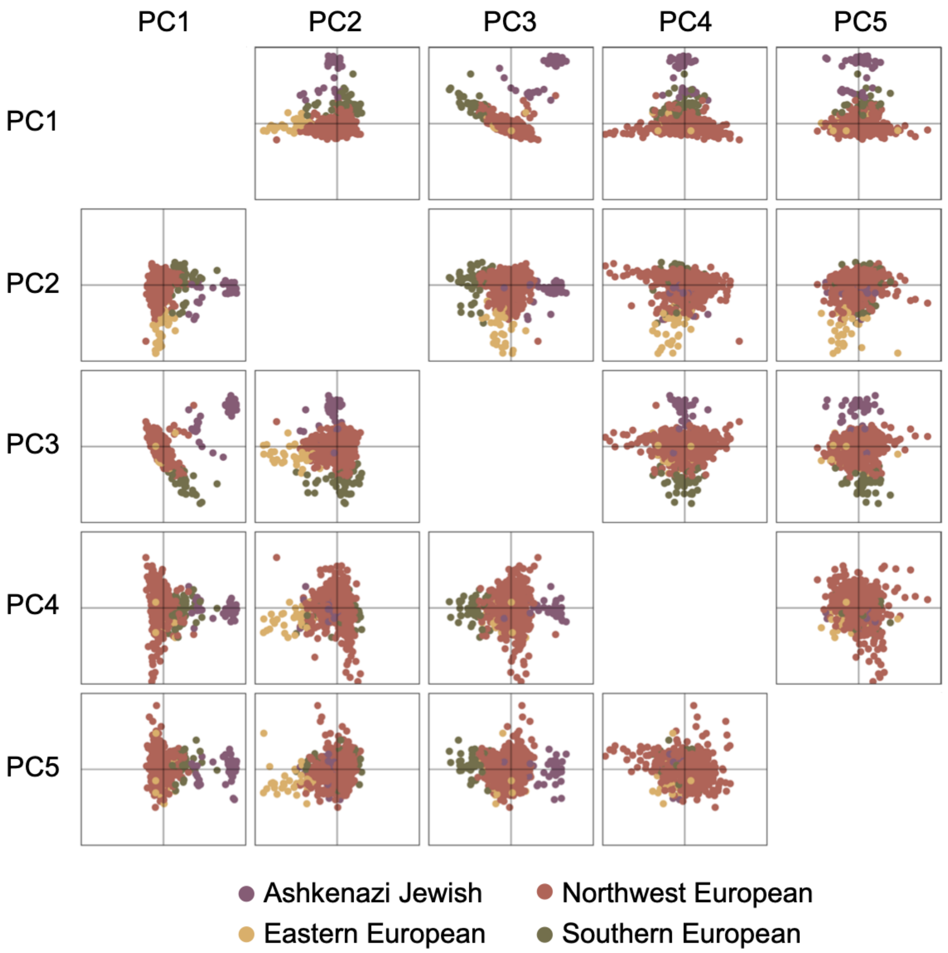

Supplementary Figure 2. Scatter matrix of the first 5 ancestry principal components (PCs) for European participants plotted from a random sampling of *n*=1,000 PIP participants. We classified individuals by their maximum genetic ancestry using 12 regional populations predicted by Ancestry Composition (Durand et al., 2014); however, only European participants are plotted here.

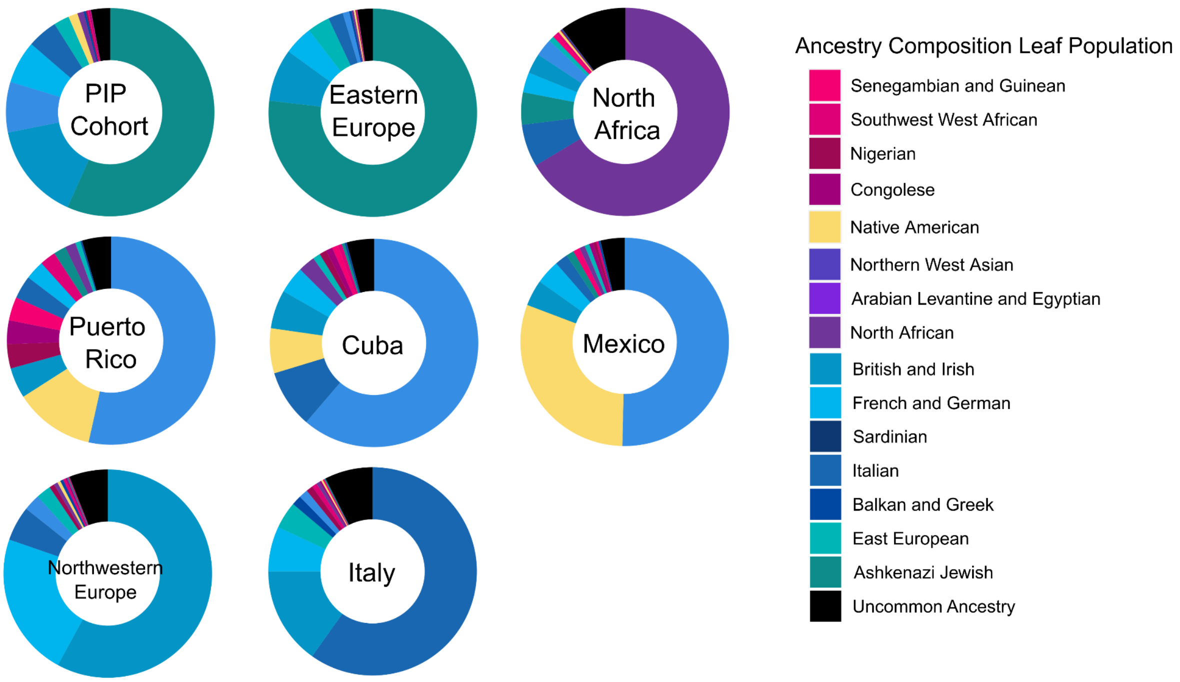

Supplementary Figure 3. Mean Ancestry Composition of the PIP cohort and LRRK2 G2019S carrier genetic groups. Averages of 23andMe Ancestry Composition populations that are present at a rate of $\geq$ 5% in the LRRK2 G2019S cohort shown in the entire PIP Cohort and each of the genetic groups determined from the entire LRRK2 G2019s cohort. All uncommon ancestry populations are binned into the broader "Uncommon Ancestry" category.

Supplementary Table 1. *Sample Sizes of Completed Follow-up Surveys for LRRK2 G2019S Carriers.*

Month	All	Older Non-Manifest	PD
0	1286	717	188
6	604 (47%)	349 (49%)	117 (62%)
12	478 (37%)	295 (41%)	103 (55%)
18	395 (31%)	234 (33%)	90 (48%)
24	335 (26%)	215 (30%)	76 (40%)
30	278 (22%)	180 (25%)	69 (37%)
36	175 (14%)	114 (16%)	34 (18%)
42	173 (13%)	112 (16%)	37 (20%)

Note. Numbers are reported as *n* (% from baseline month 0).

Supplementary Table 2. *Clinical Characteristics of the Parkinson's Impact Project Cohort at Study Entry: Additional Groups.*

**Category**	**Measure**	**All LRRK2 G2019S**	**All Non-carriers**	**Non-carrier Controls Matched to PD Participants**
Demographics	N	1286	109472	23010
	Male	40%	37%	53%
	Age (years)	54.32 (0.45)	54.81 (0.05)	69 (0.06)
	BMI	27.04 (0.17)	28.31 (0.02)	28.32 (0.04)
	European	80%	83%	89%
	Ashkenazi	43%	2%	4%
	GBA N370S	3%	1%	1%
	Education	83%	66%	69%
Motor	Tremor	21%	11%	11%
	Imbalance	26%	21%	25%
	Bradykinesia	15%	10%	15%
	Reduced Arm Swing	16%	7%	8%
Prodromal Markers	RBD	5%	3%	2%
	MCI Diagnosis	2%	1%	2%
	Depression (PHQ^)	4.56 (0.13)	4.3 (0.01)	3.01 (0.03)
	Olfactory Difficulties	9%	8%	9%
	OH	33%	27%	22%
	Constipation	5%	5%	4%
	Urinary Difficulties	26%	21%	23%
	Erectile Dysfunction	24%	25%	31%
	Daytime Somnolence	13%	13%	8%
Risk Factors	Occupational Toxins	2%	5%	6%
	Occupational PPE	27%	36%	34%
	Non-smoking	63%	59%	52%
	Non-use of Caffeine	26%	28%	25%
	Physical Inactivity	6%	8%	8%
	1° Relative with PD	27%	6%	8%
	Head Injury (with LOC)	11%	13%	14%

Note. Continuous measures are presented *M* (*SEM*); ^PHQ-9 was administered without question #9; PD=Parkinson's Disease; BMI=Body Mass Index; RBD=REM Sleep Behavior Disorder; MCI=Mild Cognitive Impairment; OH=Orthostatic Hypotension; PPE=Personal Protective Equipment; LOC=Loss of Consciousness; FDR=False Discovery Rate.

Supplementary Table 3. *Sample Sizes of Grandparent Birth Countries by Cohort.*

Grandparent Birth Country	PIP cohort count	G2019S cohort count	Total participant count
United States of America (US)	1338	6846	8650108
Russian Federation (RU)	322	1377	170338
Poland (PL)	297	1251	240349
Ukraine (UA)	172	855	124546
Puerto Rico (PR)	72	823	171435
Mexico (MX)	45	700	508441
Italy (IT)	118	697	424453
Cuba (CU)	79	533	94964
Great Britain (GB)	63	473	664248
Belarus (BY)	72	410	35353
Lithuania (LT)	93	379	47787
Germany (DE)	93	372	281918
Morocco (MA)	5	283	13235
Spain (ES)	39	238	98614
Austria (AT)	65	233	47368
Hungary (HU)	35	213	65335
Romania (RO)	40	212	55645
Algeria (DZ)	11	209	8224
Portugal (PT)	9	151	63549
Canada (CA)	38	142	215170
Brazil (BR)	27	142	69939
Latvia (LV)	34	125	14559
France (FR)	7	112	88628
Colombia (CO)	22	107	73827
Ireland (IE)	19	106	201917
Israel (IL)	11	88	10455
Dominican Republic (DO)	18	87	55910
Chile (CL)	12	84	18936
Tunisia (TN)	5	79	4705
Venezuela (VE)	9	72	25486
Greece (GR)	16	61	69298
Argentina (AR)	5	60	27097
Egypt (EG)	7	60	18312
Guatemala (GT)	5	56	30428
Moldova (MD)	5	54	7867
Peru (PE)	16	53	35662
Czechia (CZ)	9	53	37483
Syrian Arab Republic (SY)	5	49	16185
Turkey (TR)	5	49	45258
South Africa (ZA)	7	48	26125
Norway (NO)	6	42	58641
Australia (AU)	5	37	44753
Sweden (SE)	7	37	63928
Slovakia (SK)	5	32	26805
Lebanon (LB)	5	31	26321
Switzerland (CH)	8	28	29085
Philippines (PH)	5	25	106280
Netherlands (NL)	5	24	46634
Honduras (HN)	5	22	16703
Iran (IR)	5	18	46936

Uruguay (UY)	5	17	5835
Ecuador (EC)	5	15	29453
Iraq (IQ)	5	15	13244
China (CN)	6	14	129838
Panama (PA)	7	13	12510
Palestine (PS)	5	13	10489
Jamaica (JM)	5	11	42182
Nicaragua (NI)	5	11	19022
Jordan (JO)	5	11	3263
Bulgaria (BG)	5	11	17555
India (IN)	5	10	109533
Zimbabwe (ZW)	5	10	1786
Iceland (IS)	5	10	3830
Uzbekistan (UZ)	5	10	5907
Cyprus (CY)	5	10	9802
North Macedonia (MK)	5	10	6109
Kenya (KE)	5	10	2469
Estonia (EE)	5	10	5212

Note. Reported grandparent birth countries in the PIP cohort, the full LRRK2 G2019S carrier cohort, which includes the PIP cohort, and all research participants in the 23andMe database that reported their grandparents were born in the same geographic regions. To protect participant privacy, we excluded countries with small counts (n<5).

Supplementary Table 4. *Reported Grandparent Birth Countries by LRRK2 G2019S Genetic Groups.*

Genetic group	Grandparent birth country	G2019S carrier count	Total participant count	Carrier frequency
Cuba	Cuba (CU)	474	95369	0.00497
Cuba	Chile (CL)	71	19036	0.00373
Cuba	Venezuela (VE)	57	25653	0.00222
Cuba	Portugal (PT)	128	63740	0.00201
Cuba	Uruguay (UY)	10	5859	0.00171
Cuba	Brazil (BR)	111	70276	0.00158
Cuba	Spain (ES)	120	98925	0.00121
Cuba	Dominican Republic (DO)	65	56217	0.00116
Cuba	Colombia (CO)	79	74264	0.00106
Cuba	Argentina (AR)	17	27224	0.00062
Cuba	Italy (IT)	72	425699	0.00017
Cuba	France (FR)	13	88880	0.00015
Cuba	Germany (DE)	10	282663	0.00004
Cuba	United States of America (US)	189	8681625	0.00002
Cuba	Great Britain (GB)	11	666326	0.00002
East Europe	Belarus (BY)	407	35494	0.01147
East Europe	Latvia (LV)	123	14602	0.00842
East Europe	Russian Federation (RU)	1365	170856	0.00799
East Europe	Lithuania (LT)	375	47921	0.00783
East Europe	Moldova (MD)	53	7888	0.00672
East Europe	Ukraine (UA)	840	125052	0.00672
East Europe	Israel (IL)	66	10496	0.00629
East Europe	Poland (PL)	1202	241010	0.00499
East Europe	Austria (AT)	214	47451	0.00451
East Europe	Romania (RO)	205	55838	0.00367
East Europe	Hungary (HU)	208	65488	0.00318
East Europe	Tunisia (TN)	11	4717	0.00233
East Europe	Morocco (MA)	29	13286	0.00218
East Europe	South Africa (ZA)	45	26238	0.00172
East Europe	Argentina (AR)	37	27224	0.00136
East Europe	Czechia (CZ)	44	37582	0.00117
East Europe	Iraq (IQ)	13	13319	0.00098
East Europe	Germany (DE)	265	282663	0.00094
East Europe	Syrian Arab Republic (SY)	15	16238	0.00092
East Europe	Slovakia (SK)	23	26858	0.00086
East Europe	Turkey (TR)	37	45426	0.00081
East Europe	Australia (AU)	31	44830	0.00069
East Europe	Egypt (EG)	11	18405	0.0006
East Europe	Greece (GR)	40	69499	0.00058
East Europe	Canada (CA)	94	215749	0.00044
East Europe	United States of America (US)	3775	8681625	0.00043
East Europe	Switzerland (CH)	11	29143	0.00038
East Europe	Netherlands (NL)	17	46768	0.00036
East Europe	Great Britain (GB)	237	666326	0.00036
East Europe	Norway (NO)	18	58792	0.00031
East Europe	France (FR)	26	88880	0.00029
East Europe	Italy (IT)	108	425699	0.00025
East Europe	Ireland (IE)	47	202512	0.00023
East Europe	Spain (ES)	21	98925	0.00021

East Europe	Brazil (BR)	14	70276	0.0002
East Europe	Sweden (SE)	10	64080	0.00016
East Europe	Cuba (CU)	10	95369	0.0001
East Europe	Puerto Rico (PR)	17	172278	0.0001
East Europe	Mexico (MX)	12	510699	0.00002
Italy	Italy (IT)	213	425699	0.0005
Italy	United States of America (US)	144	8681625	0.00002
Mexico	Mexico (MX)	613	510699	0.0012
Mexico	Spain (ES)	30	98925	0.0003
Mexico	United States of America (US)	165	8681625	0.00002
North Africa	Algeria (DZ)	136	8243	0.0165
North Africa	Morocco (MA)	209	13286	0.01573
North Africa	Libya (LY)	21	2033	0.01033
North Africa	Tunisia (TN)	45	4717	0.00954
North Africa	Syrian Arab Republic (SY)	20	16238	0.00123
North Africa	Greece (GR)	12	69499	0.00017
North Africa	Italy (IT)	12	425699	0.00003
North Africa	Great Britain(GB)	10	666326	0.00002
North Africa	United States of America (US)	26	8681625	0
Northwestern Europe	Norway (N O)	20	58792	0.00034
Northwestern Europe	Sweden (SE)	16	64080	0.00025
Northwestern Europe	Great Britain(GB)	153	666326	0.00023
Northwestern Europe	United States of America (US)	1807	8681625	0.00021
Northwestern Europe	Italy (IT)	78	425699	0.00018
Northwestern Europe	Ireland (IE)	37	202512	0.00018
Northwestern Europe	Germany (DE)	42	282663	0.00015
Northwestern Europe	Canada (CA)	31	215749	0.00014
Northwestern Europe	Spain (ES)	14	98925	0.00014
Northwestern Europe	Brazil (BR)	10	70276	0.00014
Puerto Rico	Puerto Rico (PR)	799	172278	0.00464
Puerto Rico	Dominican Republic (DO)	18	56217	0.00032
Puerto Rico	Cuba (CU)	26	95369	0.00027
Puerto Rico	Portugal (PT)	14	63740	0.00022
Puerto Rico	Spain (ES)	21	98925	0.00021
Puerto Rico	Mexico (MX)	14	510699	0.00003
Puerto Rico	Italy (IT)	13	425699	0.00003
Puerto Rico	United States of America (US)	194	8681625	0.00002

Note. Carrier frequency is the number of grandparents reported to have been born in a country by LRRK2 G2019S carriers (G2019S carrier count) divided by the total number of grandparents reported to have been born in a country by the entire 23andMe participant database (total participant count). To protect participant privacy, we excluded countries with small counts (n<5).

Supplementary Table 5. *Symptom Prevalence Between LRRK2 G2019S PD and Non-carrier PD Adjusting for Baseline Age, Disease Duration, and Education.*

				**95% CI**				
**Domain**	**Symptom**	**Term**	***OR***	***LL***	***UL***	***SE***	***Z***	p_{FDR}
Autonomic	Constipation	Intercept	0.16	0.04	0.55	0.64	-2.87	0.017
		Symptom	0.70	0.40	1.16	0.27	-1.30	0.271
		Baseline Age	0.98	0.97	1.00	0.01	-1.79	0.144
		Education	1.71	1.16	2.61	0.21	2.63	0.037
		Disease Duration	1.04	1.01	1.06	0.01	2.62	0.011
	Erectile Dysfunction	Intercept	0.06	0.01	0.41	1.02	-2.78	0.017
		Symptom	0.76	0.48	1.21	0.24	-1.14	0.334
		Baseline Age	1.00	0.97	1.03	0.01	-0.13	0.898
		Education	1.33	0.74	2.58	0.32	0.90	0.369
		Disease Duration	1.05	1.01	1.09	0.02	2.56	0.012
	Nocturia	Intercept	0.16	0.04	0.54	0.64	-2.88	0.017
		Symptom	1.13	0.75	1.65	0.20	0.59	0.582
		Baseline Age	0.98	0.97	1.00	0.01	-1.81	0.144
		Education	1.62	1.10	2.45	0.20	2.37	0.037
		Disease Duration	1.04	1.01	1.06	0.01	2.78	0.007
	Orthostatic Hypotension	Intercept	0.17	0.05	0.63	0.66	-2.64	0.017
		Symptom	0.73	0.52	1.00	0.16	-1.97	0.073
		Baseline Age	0.98	0.97	1.00	0.01	-1.70	0.144
		Education	1.57	1.07	2.38	0.20	2.23	0.039
		Disease Duration	1.04	1.01	1.07	0.01	2.98	0.007
	Urinary Difficulties	Intercept	0.13	0.03	0.46	0.66	-3.10	0.017
		Symptom	0.88	0.64	1.20	0.16	-0.82	0.458
		Baseline Age	0.99	0.97	1.01	0.01	-1.38	0.175
		Education	1.67	1.13	2.54	0.21	2.49	0.037
		Disease Duration	1.04	1.01	1.07	0.01	3.02	0.007
Cognition	Attention/ Concentrating	Intercept	0.26	0.07	0.96	0.67	-1.99	0.048
		Symptom	0.50	0.36	0.70	0.17	-3.97	2.02E-04
		Baseline Age	0.98	0.96	1.00	0.01	-2.11	0.144
		Education	1.55	1.04	2.36	0.21	2.11	0.042
		Disease Duration	1.04	1.02	1.07	0.01	3.11	0.007
	Distracted Easily	Intercept	0.27	0.07	1.02	0.68	-1.90	0.057
		Symptom	0.51	0.36	0.71	0.17	-3.86	2.60E-04
		Baseline Age	0.98	0.96	1.00	0.01	-2.17	0.144
		Education	1.64	1.10	2.54	0.21	2.33	0.037

		Disease Duration	1.04	1.01	1.07	0.01	2.92	0.007
	MCI	Intercept	0.18	0.05	0.64	0.66	-2.60	0.018
		Symptom	0.28	0.10	0.62	0.46	-2.77	0.011
		Baseline Age	0.98	0.97	1.00	0.01	-1.75	0.144
		Education	1.48	1.00	2.24	0.20	1.91	0.059
		Disease Duration	1.04	1.01	1.07	0.01	2.98	0.007
	Multitasking	Intercept	0.25	0.07	0.91	0.67	-2.07	0.042
		Symptom	0.41	0.30	0.57	0.17	-5.23	3.65E-06
		Baseline Age	0.98	0.97	1.00	0.01	-1.73	0.144
		Education	1.52	1.02	2.33	0.21	1.97	0.054
		Disease Duration	1.04	1.01	1.07	0.01	2.85	0.007
	Task Completion	Intercept	0.23	0.06	0.83	0.67	-2.22	0.031
		Symptom	0.43	0.28	0.64	0.21	-4.00	2.02E-04
		Baseline Age	0.98	0.96	1.00	0.01	-2.08	0.144
		Education	1.72	1.15	2.67	0.22	2.52	0.037
		Disease Duration	1.04	1.01	1.07	0.01	2.87	0.007
	Understanding	Intercept	0.16	0.04	0.59	0.67	-2.72	0.017
		Symptom	0.40	0.26	0.60	0.21	-4.29	7.47E-05
		Baseline Age	0.99	0.97	1.01	0.01	-1.39	0.175
		Education	1.55	1.04	2.38	0.21	2.10	0.042
		Disease Duration	1.04	1.01	1.07	0.01	2.87	0.007
Memory	Finding the Right Word	Intercept	0.17	0.05	0.63	0.66	-2.64	0.017
		Symptom	0.67	0.48	0.93	0.17	-2.44	0.026
		Baseline Age	0.99	0.97	1.00	0.01	-1.49	0.175
		Education	1.68	1.14	2.58	0.21	2.50	0.037
		Disease Duration	1.03	1.01	1.06	0.01	2.52	0.013
	Forgetting the Date	Intercept	0.17	0.05	0.61	0.66	-2.68	0.017
		Symptom	0.41	0.27	0.60	0.20	-4.45	5.00E-05
		Baseline Age	0.99	0.97	1.00	0.01	-1.59	0.157
		Education	1.56	1.06	2.39	0.21	2.16	0.040
		Disease Duration	1.05	1.02	1.07	0.01	3.40	0.005
	Misplacing Things	Intercept	0.20	0.05	0.76	0.69	-2.34	0.025
		Symptom	0.46	0.31	0.67	0.20	-3.95	2.02E-04
		Baseline Age	0.98	0.96	1.00	0.01	-1.88	0.144
		Education	1.72	1.14	2.67	0.22	2.50	0.037
		Disease Duration	1.05	1.02	1.07	0.01	3.24	0.006
	Worsened Memory	Intercept	0.21	0.05	0.81	0.70	-2.23	0.031
		Symptom	0.56	0.40	0.79	0.17	-3.38	0.002
		Baseline Age	0.98	0.96	1.00	0.01	-1.70	0.144
		Education	1.70	1.12	2.70	0.22	2.38	0.037

		Disease Duration	1.04	1.01	1.07	0.01	2.51	0.013
Motor	Bradykinesia	Intercept	0.19	0.05	0.67	0.65	-2.55	0.019
		Symptom	0.70	0.51	0.97	0.16	-2.20	0.045
		Baseline Age	0.98	0.97	1.00	0.01	-1.74	0.144
		Education	1.60	1.09	2.42	0.20	2.30	0.037
		Disease Duration	1.04	1.01	1.07	0.01	2.91	0.007
	Postural Instability	Intercept	0.12	0.03	0.44	0.66	-3.18	0.017
		Symptom	0.96	0.69	1.36	0.17	-0.22	0.823
		Baseline Age	0.99	0.97	1.01	0.01	-1.39	0.175
		Education	1.64	1.11	2.50	0.21	2.40	0.037
		Disease Duration	1.04	1.01	1.07	0.01	2.92	0.007
	Reduced Arm Swing	Intercept	0.17	0.04	0.68	0.73	-2.45	0.020
		Symptom	0.80	0.53	1.23	0.21	-1.06	0.351
		Baseline Age	0.99	0.97	1.01	0.01	-1.42	0.175
		Education	1.71	1.13	2.68	0.22	2.44	0.037
		Disease Duration	1.03	1.00	1.06	0.01	2.16	0.031
	Tremor	Intercept	0.18	0.05	0.68	0.68	-2.49	0.019
		Symptom	0.82	0.56	1.21	0.20	-1.04	0.351
		Baseline Age	0.98	0.97	1.00	0.01	-1.73	0.144
		Education	1.56	1.06	2.36	0.20	2.19	0.040
		Disease Duration	1.04	1.01	1.06	0.01	2.77	0.007
RBD	RBD	Intercept	0.16	0.04	0.57	0.65	-2.81	0.017
		Symptom	0.31	0.18	0.51	0.26	-4.43	5.00E-05
		Baseline Age	0.99	0.97	1.00	0.01	-1.63	0.154
		Education	1.58	1.07	2.40	0.21	2.23	0.039
		Disease Duration	1.05	1.03	1.08	0.01	3.89	0.002
Smell	Olfactory Difficulties	Intercept	0.20	0.06	0.69	0.64	-2.51	0.019
		Symptom	0.43	0.31	0.59	0.17	-5.08	3.86E-06
		Baseline Age	0.98	0.97	1.00	0.01	-1.82	0.144
		Education	1.76	1.19	2.66	0.20	2.76	0.037
		Disease Duration	1.05	1.02	1.07	0.01	3.45	0.005

Note. MCI=mild cognitive impairment; RBD=REM sleep behavior disorder; OR=odds ratio; CI = confidence interval; LL=lower limit; UL=upper limit; SE=Standard error; Z=Z value, p_{FDR}=p-values adjusted for false discovery rate.

Supplementary Table 6. *Logistic Regression Coefficients and Relative Odds of PD Across LRRK2 G2019S Carriers and Non-carriers: Comparing PRS With and Without the LRRK2 Locus in All Participants.*

				**95% CI**				
**PRS**	**Carrier Status**	**Term**	***OR***	***LL***	***UL***	***SE***	***Z***	***p***
Original	Non-carrier	Intercept	0.01	0.01	0.02	0.037	-115.43	0.00E+00
		PRS	1.63	1.56	1.70	0.022	21.82	1.47E-105
		Age	1.93	1.84	2.02	0.025	26.76	1.01E-157
		Male	1.78	1.63	1.94	0.045	12.92	3.62E-38
		PC1	0.92	0.86	0.98	0.032	-2.61	0.009
		PC2	0.94	0.88	1.00	0.033	-1.86	0.062
		PC3	1.05	0.99	1.12	0.033	1.50	0.133
		PC4	0.97	0.93	1.02	0.025	-1.13	0.259
		PC5	1.01	0.96	1.08	0.029	0.37	0.712
		PC6	1.11	1.05	1.17	0.028	3.62	2.91E-04
		PC7	0.99	0.93	1.06	0.033	-0.18	0.855
		PC8	1.05	1.01	1.10	0.022	2.40	0.017
		PC9	0.97	0.92	1.02	0.028	-1.19	0.235
		PC10	0.96	0.92	1.01	0.025	-1.45	0.146
	LRRK2 G2019S	Intercept	0.19	0.15	0.24	0.127	-13.22	7.11E-40
		PRS	1.65	1.37	1.99	0.094	5.29	1.21E-07
		Age	2.44	1.99	3.04	0.108	8.28	1.23E-16
		Male	0.99	0.69	1.40	0.180	-0.08	0.935
		PC1	1.06	0.72	1.50	0.182	0.29	0.769
		PC2	0.92	0.66	1.25	0.160	-0.55	0.585
		PC3	1.07	0.75	1.56	0.182	0.40	0.692
		PC4	0.67	0.40	1.11	0.260	-1.55	0.122
		PC5	1.41	0.85	2.36	0.260	1.33	0.183
		PC6	1.19	0.78	1.84	0.220	0.80	0.421
		PC7	0.93	0.58	1.48	0.238	-0.29	0.775
		PC8	0.93	0.62	1.41	0.209	-0.34	0.732
		PC9	1.11	0.92	1.33	0.093	1.10	0.272
		PC10	0.84	0.66	1.08	0.126	-1.35	0.178
Modified	Non-carrier	Intercept	0.01	0.01	0.02	0.037	-115.46	0.00E+00
		PRS	1.62	1.55	1.69	0.022	21.62	1.26E-103
		Age	1.93	1.84	2.02	0.025	26.75	1.20E-157
		Male	1.78	1.63	1.94	0.045	12.88	5.58E-38
		PC1	0.92	0.86	0.98	0.032	-2.53	0.012
		PC2	0.94	0.88	1.00	0.033	-1.80	0.072
		PC3	1.04	0.98	1.12	0.033	1.33	0.184
		PC4	0.98	0.93	1.03	0.025	-0.86	0.388
		PC5	1.01	0.96	1.07	0.029	0.32	0.752
		PC6	1.11	1.05	1.17	0.028	3.59	3.28E-04
		PC7	1.00	0.93	1.06	0.032	-0.14	0.886
		PC8	1.05	1.01	1.10	0.022	2.35	0.019
		PC9	0.97	0.92	1.02	0.028	-1.16	0.244
		PC10	0.96	0.92	1.01	0.025	-1.51	0.132

LRRK2 G2019S	Intercept	0.19	0.15	0.24	0.126	-13.22	7.10E-40
	PRS	1.56	1.31	1.87	0.090	4.97	6.59E-07
	Age	2.44	1.99	3.04	0.108	8.27	1.35E-16
	Male	0.97	0.68	1.38	0.179	-0.15	0.884
	PC1	1.05	0.71	1.48	0.180	0.25	0.801
	PC2	0.91	0.65	1.24	0.160	-0.61	0.541
	PC3	1.07	0.74	1.54	0.181	0.35	0.725
	PC4	0.68	0.40	1.12	0.260	-1.51	0.132
	PC5	1.42	0.86	2.38	0.259	1.36	0.174
	PC6	1.23	0.80	1.89	0.219	0.94	0.345
	PC7	0.93	0.58	1.47	0.237	-0.30	0.765
	PC8	0.95	0.63	1.44	0.208	-0.25	0.799
	PC9	1.10	0.92	1.32	0.093	1.01	0.312
	PC10	0.85	0.66	1.09	0.125	-1.28	0.199

Note. Age and PCs were Z-scored prior to modeling and are interpreted in *SD* units. PRS=polygenic risk score; OR=odds ratio; CI=confidence interval; LL=lower level; UL=upper level; SE=standard error; Z=Z-value; PC=principal component.

Supplementary Table 7. *Logistic Regression Coefficients and Relative Odds of PD Across LRRK2 G2019S Carriers and Non-carriers: Comparing PRS With and Without the LRRK2 Locus in Participants with European Ancestry.*

				**95% CI**				
**PRS**	**Carrier Status**	**Term**	***OR***	***LL***	***UL***	***SE***	***Z***	***p***
Original	Non-carrier	Intercept	0.02	0.01	0.02	0.038	-109.54	0.00E+00
		PRS	1.63	1.56	1.71	0.023	21.00	6.74E-98
		Age	1.88	1.79	1.97	0.026	24.69	1.28E-134
		Male	1.80	1.64	1.97	0.047	12.59	2.33E-36
		PC1	0.99	0.95	1.04	0.021	-0.25	0.802
		PC2	0.94	0.90	0.98	0.022	-3.01	0.003
		PC3	1.08	1.03	1.13	0.024	3.10	0.002
		PC4	1.01	0.96	1.05	0.022	0.33	0.741
		PC5	0.98	0.94	1.03	0.023	-0.81	0.419
	LRRK2 G2019S	Intercept	0.21	0.16	0.27	0.134	-11.76	6.21E-32
		PRS	1.76	1.44	2.16	0.103	5.49	3.95E-08
		Age	2.54	2.04	3.22	0.117	7.99	1.31E-15
		Male	0.95	0.65	1.38	0.191	-0.28	0.780
		PC1	0.85	0.61	1.17	0.165	-0.98	0.327
		PC2	0.94	0.78	1.15	0.100	-0.60	0.549
		PC3	1.10	0.80	1.57	0.170	0.58	0.560
		PC4	1.06	0.88	1.29	0.098	0.63	0.530
		PC5	1.09	0.90	1.32	0.098	0.85	0.396
Modified	Non-carrier	Intercept	0.02	0.01	0.02	0.038	-109.56	0.00E+00
		PRS	1.63	1.56	1.71	0.023	20.83	2.10E-96
		Age	1.88	1.79	1.97	0.026	24.69	1.39E-134
		Male	1.79	1.64	1.97	0.047	12.56	3.33E-36
		PC1	1.00	0.96	1.04	0.021	-0.01	0.989
		PC2	0.94	0.90	0.98	0.022	-3.01	0.003
		PC3	1.08	1.03	1.13	0.024	3.06	0.002
		PC4	1.01	0.96	1.05	0.022	0.31	0.758
		PC5	0.98	0.94	1.02	0.023	-0.84	0.400
	LRRK2 G2019S	Intercept	0.21	0.16	0.27	0.133	-11.72	1.04E-31
		PRS	1.65	1.36	2.00	0.098	5.10	3.39E-07
		Age	2.53	2.03	3.21	0.117	7.98	1.45E-15
		Male	0.93	0.64	1.35	0.190	-0.39	0.698
		PC1	0.86	0.62	1.19	0.165	-0.90	0.367
		PC2	0.93	0.77	1.14	0.100	-0.75	0.452
		PC3	1.15	0.84	1.63	0.169	0.85	0.398
		PC4	1.07	0.88	1.30	0.098	0.67	0.500
		PC5	1.08	0.90	1.31	0.097	0.83	0.407

Note. Age and PCs were Z-scored prior to modeling and are interpreted in *SD* units. PRS=polygenic risk score; OR=odds ratio; CI=confidence interval; LL=lower level; UL=upper level; SE=standard error; Z=Z-value; PC=principal component.

Supplementary Table 8. *Logistic Regression Coefficients and Relative Odds of PD Across Non-Genetic Risk Factors and Education in LRRK2 G2019S Carriers and Non-carriers.*

			**95% *CI***				
**LRRK2 G2019S**	**Term**	***OR***	***LL***	***UL***	***SE***	***Z***	***p***
Carriers	Male	1.19	0.84	1.69	0.18	1	3.19E-01
	Non-smoking	1.2	0.84	1.72	0.18	0.98	3.28E-01
	Education	1.15	0.71	1.94	0.26	0.55	5.80E-01
	Occupational Toxin Exposure	1.67	0.52	4.65	0.55	0.94	3.45E-01
	Caffeine Non-use	1.62	1.11	2.35	0.19	2.54	1.11E-02
	Previous Head Injury with LOC	1.14	0.65	1.92	0.27	0.49	6.25E-01
Non-carriers	Male	1.92	1.73	2.12	0.05	12.59	2.33E-36
	Non-smoking	1.17	1.06	1.3	0.05	2.97	2.96E-03
	Education	1.34	1.2	1.51	0.06	4.96	6.99E-07
	Occupational Toxin Exposure	2.1	1.77	2.46	0.08	8.84	9.53E-19
	Caffeine Non-use	1.49	1.34	1.66	0.05	7.39	1.46E-13
	Previous Head Injury with LOC	1.21	1.05	1.38	0.07	2.71	6.73E-03

Note. CI=confidence interval, LOC=loss of consciousness. Participants were ≥ 40 years of age. *n*=162 LRRK2 G2019S PD; *n*=694 LRRK2 G2019S non-manifest; *n*=1,589 non-carrier PD; *n*=73,791 non-carrier controls.

To examine the role of non-genetic factors, we regressed PD status against the same risk factors used in AFT modeling separately for *LRRK2 G2019S* carriers and non-carriers ≥ 40 years of age. We observed that *LRRK2 G2019S* participants with PD versus those without PD reported similar frequencies across risk factors, with the exception of caffeine in which *LRRK2 G2019S* PD cases reported less caffeine use as compared to *LRRK2 G2019S* non-manifest individuals. In contrast, non-carrier PD reported more risk factors than non-carrier controls, including significantly higher occupational toxin exposure and a higher frequency of previous head injuries. Non-carriers with PD were also less likely to be smokers and consume caffeine compared to non-carrier controls.

Supplementary Table 9. *Logistic Regression Coefficients and Relative Odds of PD Across Low, Intermediate, and High Modified PRS between LRRK2 G2019S Carriers and Non-carriers in All Participants.*

		**95% CI**				
**Term**	***OR***	***LL***	***UL***	***SE***	***Z***	***p***
Intercept	0.01	0.01	0.02	0.043	-99.92	0.00E+00
Non-carrier Low	0.63	0.55	0.72	0.067	-6.93	4.08E-12
Non-carrier Inter.	1.00					
Non-carrier High	1.95	1.78	2.14	0.048	13.98	2.03E-44
LRRK2 G2019S Low	6.06	3.71	9.49	0.239	7.55	4.24E-14
LRRK2 G2019S Inter.	9.09	6.82	11.99	0.144	15.36	2.94E-53
LRRK2 G2019S High	16.87	12.55	22.55	0.149	18.92	7.73E-80
Age	1.95	1.86	2.05	0.024	28.04	5.76E-173
Male	1.70	1.56	1.85	0.043	12.36	4.23E-35
PC1	0.92	0.87	0.98	0.031	-2.50	0.012
PC2	0.94	0.88	1.00	0.033	-1.80	0.072
PC3	1.05	0.98	1.12	0.032	1.38	0.167
PC4	0.97	0.92	1.02	0.026	-1.04	0.297
PC5	1.01	0.96	1.08	0.030	0.45	0.654
PC6	1.10	1.04	1.16	0.027	3.44	0.001
PC7	0.99	0.93	1.06	0.032	-0.19	0.847
PC8	1.05	1.01	1.10	0.022	2.35	0.019
PC9	0.97	0.92	1.02	0.027	-1.05	0.296
PC10	0.96	0.92	1.01	0.024	-1.50	0.132

Note. Groups were determined by modified PRS percentile ranges: Low (1-25%), Intermediate (25-75%), and High (75-100%); non-carriers with Intermediate PRS served as the reference group; Age and PCs were Z-scored prior to modeling and are interpreted in *SD* units; PRS=polygenic risk score; Inter.=Intermediate; OR=odds ratio; SE=standard error; Z=Z-value; CI=confidence interval; LL=lower level; UL=upper level; Inter.=intermediate; PC=principal component.

Supplementary Table 10. *Logistic Regression Coefficients and Relative Odds of PD Across Low, Intermediate, and High Modified PRS between LRRK2 G2019S Carriers and Non-carriers in European Participants.*

		**95% CI**				
**Term**	***OR***	***LL***	***UL***	***SE***	***Z***	***p***
Intercept	0.02	0.01	0.02	0.044	-94.41	0.00E+00
Non-carrier Low	0.64	0.56	0.74	0.070	-6.29	3.10E-10
Non-carrier Inter.	1.00					
Non-carrier High	2.01	1.82	2.21	0.050	13.95	2.98E-44
LRRK2 G2019S Low	5.53	3.17	9.14	0.269	6.37	1.89E-10
LRRK2 G2019S Inter.	8.80	6.49	11.83	0.153	14.21	7.93E-46
LRRK2 G2019S High	17.17	12.55	23.35	0.158	17.96	3.99E-72
Age	1.91	1.82	2.00	0.025	25.97	1.11E-148
Male	1.71	1.57	1.87	0.045	11.99	4.05E-33
PC1	0.99	0.95	1.04	0.021	-0.24	0.812
PC2	0.94	0.90	0.98	0.022	-2.87	0.004
PC3	1.08	1.03	1.13	0.024	3.14	0.002
PC4	1.01	0.97	1.05	0.022	0.36	0.722
PC5	0.99	0.94	1.03	0.022	-0.68	0.498

Note. Groups were determined by modified PRS percentile ranges: Low (1-25%), Intermediate (25-75%), and High (75-100%); non-carriers with Intermediate PRS served as the reference group; Age and PCs were Z-scored prior to modeling and are interpreted in *SD* units; PRS=polygenic risk score; Inter.=Intermediate; OR=odds ratio; SE=standard error; Z=Z-value; CI=confidence interval; LL=lower level; UL=upper level; Inter.=intermediate; PC=principal component.

Supplementary Table 11. *Logistic Regression Coefficients and Relative Odds of PD Across Deciles of Polygenic Risk Score in All Participants.*

		**95% CI**				
**Term**	***OR***	***LL***	***UL***	***SE***	***Z***	***p***
Intercept	0.01	0.01	0.01	0.083	-52.52	0.00E+00
Non-carrier - 1	0.57	0.44	0.73	0.128	-4.40	1.11E-05
Non-carrier - 2	0.82	0.66	1.03	0.116	-1.67	0.095
Non-carrier - 3	0.76	0.60	0.95	0.119	-2.35	0.019
Non-carrier - 4	0.98	0.79	1.22	0.111	-0.16	0.872
Non-carrier - 5	1.00					
Non-carrier - 6	1.10	0.89	1.36	0.109	0.89	0.375
Non-carrier - 7	1.34	1.10	1.65	0.104	2.84	0.005
Non-carrier - 8	1.53	1.25	1.86	0.101	4.18	2.98E-05
Non-carrier - 9	1.84	1.52	2.23	0.098	6.19	6.04E-10
Non-carrier - 10	2.92	2.45	3.51	0.092	11.62	3.16E-31
LRRK2 G2019S - 1	4.90	1.96	10.54	0.423	3.76	1.71E-04
LRRK2 G2019S - 2	8.71	4.04	17.06	0.364	5.95	2.61E-09
LRRK2 G2019S - 3	8.93	4.38	16.92	0.342	6.40	1.59E-10
LRRK2 G2019S - 4	6.49	2.89	13.09	0.381	4.91	9.17E-07
LRRK2 G2019S - 5	11.38	6.38	19.45	0.283	8.59	8.40E-18
LRRK2 G2019S - 6	13.45	7.82	22.35	0.267	9.74	2.05E-22
LRRK2 G2019S - 7	8.38	4.45	14.87	0.306	6.95	3.73E-12
LRRK2 G2019S - 8	10.98	6.34	18.30	0.269	8.90	5.58E-19
LRRK2 G2019S - 9	18.71	11.58	29.71	0.240	12.22	2.53E-34
LRRK2 G2019S - 10	24.69	15.97	37.80	0.219	14.61	2.33E-48
Age	1.95	1.86	2.05	0.024	27.99	2.22E-172
Male	1.71	1.57	1.86	0.043	12.46	1.22E-35
PC1	0.93	0.87	0.98	0.031	-2.39	0.017
PC2	0.94	0.88	1.00	0.033	-1.82	0.068
PC3	1.04	0.98	1.11	0.032	1.31	0.192
PC4	0.97	0.92	1.02	0.026	-1.10	0.271
PC5	1.01	0.96	1.08	0.030	0.45	0.651
PC6	1.10	1.05	1.16	0.027	3.58	3.49E-04
PC7	1.00	0.93	1.06	0.032	-0.13	0.900
PC8	1.05	1.00	1.09	0.022	2.12	0.034
PC9	0.97	0.92	1.03	0.027	-0.99	0.323
PC10	0.96	0.92	1.01	0.024	-1.67	0.096

Note. Numbers to the right of groups indicate decile; non-carriers at the 5th decile of PRS served as the reference group; Age and PCs were Z-scored prior to modeling and are interpreted in *SD* units; OR=odds ratio; CI=confidence interval; LL=lower level; UL=upper level; SE=standard error; Z=Z-value; PC=principal component.

Supplementary Table 12. *Logistic Regression Coefficients and Relative Odds of PD Across Deciles of Polygenic Risk Score in European Participants.*

		**95% CI**				
**Term**	***OR***	***LL***	***UL***	***SE***	***Z***	***p***
Intercept	0.01	0.01	0.02	0.086	-49.57	0.00E+00
Non-carrier - 1	0.55	0.42	0.72	0.135	-4.39	1.12E-05
Non-carrier - 2	0.83	0.66	1.05	0.120	-1.53	0.126
Non-carrier - 3	0.73	0.57	0.93	0.124	-2.49	0.013
Non-carrier - 4	0.98	0.78	1.23	0.115	-0.18	0.861
Non-carrier - 5	1.00					
Non-carrier - 6	1.08	0.86	1.35	0.113	0.67	0.501
Non-carrier - 7	1.27	1.03	1.58	0.109	2.21	0.027
Non-carrier - 8	1.43	1.16	1.76	0.106	3.38	0.001
Non-carrier - 9	1.88	1.54	2.30	0.101	6.21	5.21E-10
Non-carrier - 10	2.95	2.45	3.57	0.096	11.32	1.05E-29
LRRK2 G2019S - 1	4.10	1.38	9.84	0.492	2.87	0.004
LRRK2 G2019S - 2	8.38	3.51	17.80	0.409	5.19	2.05E-07
LRRK2 G2019S - 3	7.59	3.49	15.09	0.370	5.47	4.39E-08
LRRK2 G2019S - 4	6.15	2.73	12.44	0.383	4.74	2.11E-06
LRRK2 G2019S - 5	12.40	6.75	21.88	0.299	8.43	3.53E-17
LRRK2 G2019S - 6	12.78	7.20	21.84	0.282	9.05	1.49E-19
LRRK2 G2019S - 7	8.51	4.39	15.54	0.321	6.68	2.37E-11
LRRK2 G2019S - 8	7.76	4.11	13.82	0.308	6.66	2.82E-11
LRRK2 G2019S - 9	18.09	11.06	29.11	0.246	11.76	6.49E-32
LRRK2 G2019S - 10	27.11	17.11	42.66	0.233	14.19	1.10E-45
Age	1.91	1.82	2.00	0.025	25.92	4.05E-148
Male	1.73	1.58	1.88	0.045	12.10	1.12E-33
PC1	0.99	0.95	1.04	0.022	-0.30	0.761
PC2	0.93	0.90	0.98	0.022	-3.12	0.002
PC3	1.07	1.03	1.13	0.024	3.04	0.002
PC4	1.01	0.97	1.05	0.022	0.46	0.646
PC5	0.98	0.94	1.03	0.022	-0.69	0.493

Note. Numbers to the right of groups indicate decile; non-carriers at the 5th decile of PRS served as the reference group; Age and PCs were Z-scored prior to modeling and are interpreted in *SD* units; OR=odds ratio; CI=confidence interval; LL=lower level; UL=upper level; SE=standard error; Z=Z-value; PC=principal component.

Supplementary Table 13. Linear Regression Coefficients of the Association Between Age of PD Diagnosis and Modified PRS in All PD Participants and LRRK2 G2019S PD.

			95% *CI*				
**Model**	**Term**	***b***	***LL***	***UL***	***SE***	***Z***	***p***
All PD	Intercept	60.82	59.98	61.65	0.42		
	Sex	0.84	0.02	1.66	0.42	2.01	0.044
	PC1	-0.95	-1.64	-0.26	0.35	-2.70	7.09E-03
	PC2	0.52	-0.17	1.22	0.35	1.47	0.141
	PC3	0.16	-0.53	0.84	0.35	0.45	0.655
	PC4	-0.19	-0.66	0.28	0.24	-0.80	0.421
	PC5	0.29	-0.31	0.88	0.30	0.95	0.342
	PC6	0.42	-0.18	1.02	0.31	1.36	0.173
	PC7	0.10	-0.69	0.89	0.40	0.25	0.805
	PC8	0.68	0.28	1.09	0.21	3.30	9.74E-04
	PC9	-0.62	-1.21	-0.02	0.30	-2.04	0.041
	PC10	0.05	-0.46	0.56	0.26	0.20	0.844
	Modified PRS	-1.25	-1.98	-0.52	0.37	-3.35	8.13E-04
	LRRK2 G2019S	-3.75	-5.51	-1.99	0.90	4.17	3.12E-05
	Modified PRS * LRRK2 G2019S	0.04	-1.41	1.50	0.74	-0.06	0.954
LRRK2 G2019S PD	Intercept	60.53	59.27	61.80	0.64		
	Sex	1.45	-1.15	4.05	1.32	1.10	0.271
	PC1	-2.16	-7.14	2.81	2.52	-0.86	0.392
	PC2	2.98	-2.20	8.15	2.62	1.14	0.258
	PC3	1.15	-2.09	4.40	1.64	0.70	0.484
	PC4	1.14	-0.95	3.23	1.06	1.08	0.284
	PC5	-0.26	-3.97	3.45	1.88	-0.14	0.891
	Modified PRS	-1.35	-2.67	-0.02	0.67	-2.00	0.047

Note. Modified PRS=polygenic risk score without LRRK2 locus; PCs and Modified PRS are in *SD* units and were re-centered; sex and LRRK2 G2019S status were effects coded, therefore, the intercept reflects the grand mean of PD diagnosis age at average value of covariates. *n*=1,927 non-carrier PD; *n*=189 LRRK2 G2019S PD.

References

Aguet, F., Barbeira, A. N., Bonazzola, R., Brown, A., Castel, S. E., Jo, B., Kasela, S., Kim-Hellmuth, S., Liang, Y., Oliva, M., Parsana, P. E., Flynn, E., Fresard, L., Gaamzon, E. R., Hamel, A. R., He, Y., Hormozdiari, F., Mohammadi, P., Muñoz-Aguirre, M., … Lappalainen, T. (2019). *The GTEx Consortium atlas of genetic regulatory effects across human tissues* (p. 787903). bioRxiv. https://doi.org/10.1101/787903

Auton, A., Abecasis, G. R., Altshuler, D. M., Durbin, R. M., Abecasis, G. R., Bentley, D. R., Chakravarti, A., Clark, A. G., Donnelly, P., Eichler, E. E., Flicek, P., Gabriel, S. B., Gibbs, R. A., Green, E. D., Hurles, M. E., Knoppers, B. M., Korbel, J. O., Lander, E. S., Lee, C., … National Eye Institute, N. (2015). A global reference for human genetic variation. *Nature*, *526*(7571), Article 7571. https://doi.org/10.1038/nature15393

Barton, A. R., Sherman, M. A., Mukamel, R. E., & Loh, P.-R. (2021). Whole-exome imputation within UK Biobank powers rare coding variant association and fine-mapping analyses. *Nature Genetics*, *53*(8), Article 8. https://doi.org/10.1038/s41588-021-00892-1

Bergström, A., McCarthy, S. A., Hui, R., Almarri, M. A., Ayub, Q., Danecek, P., Chen, Y., Felkel, S., Hallast, P., Kamm, J., Blanché, H., Deleuze, J.-F., Cann, H., Mallick, S., Reich, D., Sandhu, M. S., Skoglund, P., Scally, A., Xue, Y., … Tyler-Smith, C. (2020). Insights into human genetic variation and population history from 929 diverse genomes. *Science*, *367*(6484), eaay5012. https://doi.org/10.1126/science.aay5012

Browning, B. L., Zhou, Y., & Browning, S. R. (2018). A One-Penny Imputed Genome from Next-Generation Reference Panels. *The American Journal of Human Genetics*, *103*(3), 338–348. https://doi.org/10.1016/j.ajhg.2018.07.015

Durand, E. Y., Do, C. B., Mountain, J. L., & Macpherson, J. M. (2014). *Ancestry Composition: A Novel, Efficient Pipeline for Ancestry Deconvolution* (p. 010512). bioRxiv. https://doi.org/10.1101/010512

Hartwig, F. P., Davies, N. M., Hemani, G., & Davey Smith, G. (2016). Two-sample Mendelian

randomization: Avoiding the downsides of a powerful, widely applicable but potentially fallible technique. *International Journal of Epidemiology*, *45*(6), 1717–1726. https://doi.org/10.1093/ije/dyx028

Li, H., Bloom, J. M., Farjoun, Y., Fleharty, M., Gauthier, L., Neale, B., & MacArthur, D. (2018). A synthetic-diploid benchmark for accurate variant-calling evaluation. *Nature Methods*, *15*(8), Article 8. https://doi.org/10.1038/s41592-018-0054-7

Mallick, S., Li, H., Lipson, M., Mathieson, I., Gymrek, M., Racimo, F., Zhao, M., Chennagiri, N., Nordenfelt, S., Tandon, A., Skoglund, P., Lazaridis, I., Sankararaman, S., Fu, Q., Rohland, N., Renaud, G., Erlich, Y., Willems, T., Gallo, C., … Reich, D. (2016). The Simons Genome Diversity Project: 300 genomes from 142 diverse populations. *Nature*, *538*(7624), Article 7624. https://doi.org/10.1038/nature18964

Nalls, M. A., Blauwendraat, C., Vallerga, C. L., Heilbron, K., Bandres-Ciga, S., Chang, D., Tan, M., Kia, D. A., Noyce, A. J., Xue, A., Bras, J., Young, E., von Coelln, R., Simón-Sánchez, J., Schulte, C., Sharma, M., Krohn, L., Pihlstrøm, L., Siitonen, A., … Zhang, F. (2019). Identification of novel risk loci, causal insights, and heritable risk for Parkinson's disease: A meta-analysis of genome-wide association studies. *The Lancet Neurology*, *18*(12), 1091–1102. https://doi.org/10.1016/S1474-4422(19)30320-5
